# Conformational Selection of α-Synuclein Tetramers at Biological Interfaces

**DOI:** 10.1021/acs.jcim.4c01459

**Published:** 2024-10-08

**Authors:** Shayon Bhattacharya, Liang Xu, Lily Arrué, Tim Bartels, Damien Thompson

**Affiliations:** † Department of Physics, Bernal Institute, University of Limerick, Limerick V94 T9PX, Ireland; ‡ UK Dementia Research Institute, University College London, London WC1E6BT, U.K.

## Abstract

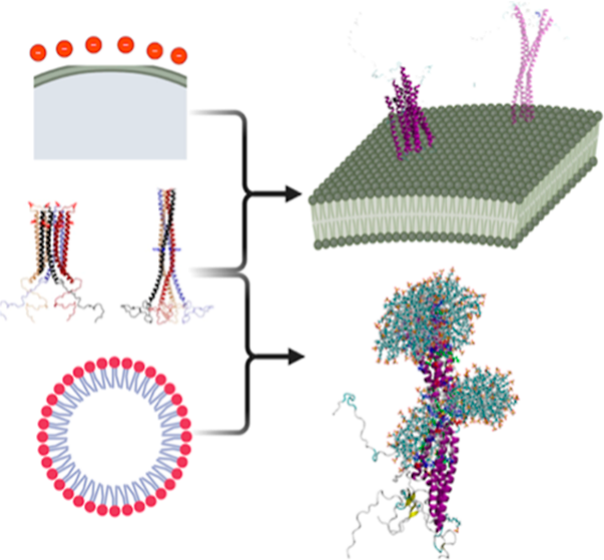

Controlling the polymorphic assemblies of α-synuclein (αS) oligomers is crucial to reroute toxic protein aggregation implicated in Parkinson’s disease (PD). One potential mediator is the interaction of αS tetramers with cell membranes, which may regulate the dynamic balance between aggregation-prone disordered monomers and aggregation-resistant helical tetramers. Here, we model diverse tetramer–cell interactions and compare the structure–function relations at the supramolecular–biological interface with available experimental data. The models predict preferential interaction of compact αS tetramers with highly charged membrane surfaces, which may further stabilize this aggregation-resistant conformer. On moderately charged membranes, extended structures are preferred. In addition to surface charge, curvature influences tetramer thermodynamic stability and aggregation, with potential for selective isolation of tetramers *via* regio-specific interactions with strongly negatively charged micelles that screen further aggregation. Our modeling data set highlights diverse beneficial nano–bio interactions to redirect biomolecule assembly, supporting new therapeutic approaches for PD based on lipid-mediated conformational selection and inhibition.

## Introduction

The 140-residue α-synuclein (αS) protein is a key biomarker of Parkinson’s disease (PD).^1^ αS is the major component of Lewy bodies and Lewy neurites in PD- and LB-related dementia and of neuronal and glial cytoplasmic inclusions in multiple system atrophy.^1,2^ αS protein may self-assemble *via* the central hydrophobic non-amyloid-β component (NAC) region (residues 61–95), which is crucial for defining the fold preference and so polymorphism of αS fibrils.^3^ Abnormal aggregation of αS can form neurotoxic β-sheet-rich oligomers that may nucleate and polymerize into insoluble amyloid fibrils via several distinct mechanisms.^4^ Abundant in presynaptic terminals, αS is intrinsically disordered^5,6^ and can adopt a compact or an extended helical conformation upon binding to cells *via* its N-terminal (residues 1–60) membrane-binding domain.^7^ The membrane binding affinity of the αS monomer is found to vary depending on membrane lipid composition, curvature, and local concentration of αS.^8^ A folded αS helical tetramer likely exists in dynamic equilibrium with disordered αS monomers natively, with the population balance dependent on interaction with various cell types and other biochemical cues.^9,10^ Known familial mutations of αS can destabilize the tetramer and shift the dynamic equilibrium back toward monomers, thus disrupting normal αS homeostasis and making them more aggregation-prone.^11,12^ On the other hand, the degree of helicity in the αS monomers may influence their tendency to form a folded helical tetramer instead of neurotoxic oligomers.^13−16^

While a broad range of nanostructure sizes and shapes contribute to the full population of αS in solution, the existence of a soluble helically folded αS tetramer has been independently confirmed by several groups.^17−21^ Structural characterization together with better understanding of supramolecular organization and thermodynamic stabilization of aggregation-impeding helical tetramers may contribute to development of anti-PD drugs.^22^ Experimental studies show that cellular membrane-associated αS adopts an α-helical conformation, while cytosolic αS is unfolded,^7^ highlighting diverse fold propensities and interactions of helical αS on cell membranes. In addition, αS monomers may exist as helical intermediates in solution (specifically, cytosol) through transient interaction with lipid interfaces.^23^ On the other hand, membrane-bound assembly of large helical multimers of αS may disrupt neuronal signaling by blocking protein–vesicle interactions at the synapse.^24^ This multifactorial effect makes it challenging to precisely map the interactions of αS with biological surfaces and their influence on neurotoxicity through experiments alone. In particular, the dynamic equilibrium between heterogeneous biological surface-bound monomers and multimers could couple with partition of the intrinsically disordered αS monomers and helically folded tetramers in the intracellular environment.^22,25^

In the present work, we investigate the relationship between conformational preference of aggregation-resistant preformed helical αS tetramers and their interactions with different kinds of surfaces and environments, as a further step toward understanding and ultimately targeting αS neurotoxicity. We map the molecular level internal packing interactions in the oligomers and the thermodynamic driving forces for the formation of two αS tetramer conformations with different helical continuity (compact and extended), stabilized by interactions with heterogeneous biological surfaces (flat lipid bilayer membranes and spherical micelles, with varying charge distributions) as predicted through molecular dynamics (MD) simulations. The models predict extended-shaped conformations that could potentially reroute self-assembly away from amyloidogenesis. Predicted binding profiles of αS tetramers with differently charged micelles show the formation of multiple, specific, long-lived complexes that resculpt the tetramer internal packing structure and so recalibrate the population balance of αS species. In a putative protective role in mediating neurodegeneration through rerouted nanobio interactions, the micelles may potentially seed a lipid corona that coats and screens further αS aggregation. As discussed below, our models serve to clarify several aspects of αS aggregation that were inferred from previous experiments and provide directly testable experimental hypotheses for future investigations.

## Materials and Methods

### Tetramer Structures

The starting structure of a compact helical αS tetramer was taken from previous work, which predicted that the multimer assembles *via* hydrophobic packing of the NAC regions.^26,27^ To construct the extended helical αS tetramer, the experimentally determined membrane-bound 11/3 (3 helical turns per 11 residues) helical αS (residues 9–89)^28^ was used to build the full-length helical monomer with added disordered C-terminus (residues 90–140) and proximal N-terminus (residues 1–8). Starting from a pool of ten extended helical monomer conformations bound to a lipid bilayer as obtained from the study by Jao et al.,^28^ we selected the extended helical monomer that showed least structural deviation from tetrabrachion, an ideal coiled coil symmetric tetramer (Note S1, Figure S1). To identify the optimal curvature of the extended helices in tetramers that could potentially bind membrane surfaces, we constructed four different extended helical tetramer models (Note S2). From the dynamics of the models in aqueous solution, we selected two representative, diverse extended helical tetramers (named Extended I and II; see Table S1). In model Extended I, each helical monomer within the tetramer was intertwined to maximize the protein–protein contacts without focusing on optimizing the hydrophobic contacts in the NAC region. In model Extended II, the hydrophobic core of the NAC region of each monomer was aligned to maximize the hydrophobic contacts, similar to the monomeric NAC arrangement in the compact tetramer. All tetramer constructs were built using the ZDOCK^29^ server.

### Lipid Bilayers

Four different types of lipid bilayers were used to investigate tetramer adsorption on the membrane. We modeled two types of anionic lipid bilayers: first, a strongly negatively charged homogeneous membrane composed of 1000 negatively charged POPS (1-palmitoyl-2-oleoyl-*sn*-*glycero*-3-phospho-l-serine) lipids, with 500 POPS lipid molecules in each leaflet, and, second, a moderately negatively charged ternary lipid mixture composed of DOPC (1,2-dioleoyl-*sn*-*glycero*-3-phosphocholine)/DOPE (1,2-dioleoyl-*sn*-*glycero*-3-phosphoethanolamine)/DOPS (1,2-dioleoyl-*sn*-*glycero*-3-phospho-l-serine) lipids in a ratio of 2:5:3, with 500 mixed lipids in each leaflet. These two anionic membranes are known to bind αS through their acidic PS (phosphatidylserine) headgroup and their chemical compositions reflect the primary structure of inner plasma membrane and synaptic vesicles.^24,30−32^ We modeled two neutral ternary lipid bilayer mixtures. The first is composed of POPC (1-palmitoyl-2-oleoyl-*sn*-*glycero*-3-phosphocholine)/CHL (cholesterol)/PSM (*N*-palmitoyl-d-*erythro*-sphingosylphosphorylcholine). Second is POPE (1-palmitoyl-2-oleoyl-*sn*-*glycero*-3-phosphoethanolamine)/CHL/PSM. Both neutral membranes have their component lipids in a 2:1:1 molar ratio and have 500 mixed lipids per leaflet. These two sphingolipid-based mixed neutral membrane types are major constituents of detergent-resistant lipid raft microdomains^33^ in the outer plasma membrane that are known to strongly associate with αS and ensure its localization on synaptic vesicles^34^ (for which α-synuclein is named). In particular, the alteration of raft microdomains of sphingomyelin (a type of sphingolipid found in animal cell membranes, especially in the membranous myelin sheath that surrounds some nerve cell axons) may be associated with αS toxicity in PD.^35,36^ Note that the magnitude of the net negative charge per lipid scales from −1*e* in the POPS lipid bilayer to −0.3*e* in the DOPC/DOPE/DOPS lipid bilayer and from 0*e* in the POPC/CHL/PSM and POPE/CHL/PSM lipid bilayers. The starting structure of each lipid bilayer was generated using the CHARMM-GUI membrane builder.^37,38^

### Anionic Micelles

To complement our differently charged anionic lipid membrane models, we designed anionic spherical surfactant micelle nanoparticles (see below for composition of micelles) using the CHARMM-GUI micelle builder^39,40^ and let them freely interact with the compact helical and extended helical models of αS tetramers. The two designed anionic micelle structures used in this study are (1) strongly negatively charged micelle [−1.0*e*/molecule] composed of 180 sodium dodecyl sulfate (SDS) molecules (closely mimicking the POPS membrane) and (2) a moderately negatively charged micelle [−0.3*e*/molecule], composed of 100 molecules of mixed surfactant molecules, glycerol monostearate 2-isomer (GMS2)/*N*-tridecylphosphocholine (FOS16)/2,3 dilauroyl-d-*glycero*-1-phosphatidyl-glycerol (LMPG) in a 2:5:3 ratio (to mimic the DOPC/DOPE/DOPS membrane). To evaluate the tetramer–micelle interactions, the micelle nanoparticles were positioned initially ∼0.5–1 nm from the tetramer in the compact and extended helical structures (see Figures S2–S5, for a total of eight different starting configurations; see Table S2). We note that the root-mean-square deviation (rmsd) and the fraction of native contacts equilibrated within the first ∼150 ns of dynamics (Figure S6) for each complex. The tetramer internal hydrogen bonds and the secondary structure were preserved (Figures S7–S10) in all MD production runs.

### MD Simulations

MD simulations were performed using GROMACS-2018.4 software.^41^ The αS tetramer, lipid bilayer, and micelles were represented by CHARMM force field parameters, CHARMM36m^42^ and CHARMM36,^43^ respectively. The tetramer was placed on the membrane surface (*xy*-plane) at a minimum distance of either 15 Å (POPS and DOPC/DOPE/DOPS) or 5 Å (POPS, DOPC/DOPE/DOPS, POPC/CHL/PSM and POPE/CHL/PSM) above the lipid bilayer. The tetramer–membrane complex was solvated by filling the area above and below the membrane, with water molecules represented by the modified TIP3P water model,^42^ creating a >20 Å thick water layer above the protein and below the membrane to mimic bulk solvation in the *z*-plane. Each simulation cell was neutralized by adding the appropriate number of counterions. After 5000 steps of energy minimization, each system was equilibrated over six consecutive steps (100 ps each step), with the values of the force constants of position and dihedral restraints of lipids gradually decreased from 1000 to 0 (the unit for position and dihedral restraints are kJ/(mol nm^2^) and kJ/(mol rad^2^), respectively), and the force constant values of protein backbone and side chain atoms decreased from 4000 to 0 and 2000 to 0, respectively. During equilibration, the Berendsen^44^ thermostat and barostat were applied to maintain the temperature at 310 K and pressure at 1 atm. Semi-isotropic pressure coupling was applied to allow the lipid bilayer to fluctuate in the *xy*-plane independently of the *z*-axis.

Eight different starting conformations of tetramer-micelle systems were designed; four systems with two types of micelles (SDS and GMS2/FOS16/LMPG micelles) below and to the side of compact (BSC_SDS and BSC_MIXED) and extended (BSE_SDS and BSE_MIXED) tetramers and the other four with micelle orientations above the top and the side of the tetramers (TSC_SDS, TSC_MIXED, TSE_SDS, and TSE_MIXED). For the production runs, the Nose–Hoover thermostat^45^ and Parrinello–Rahman barostat^46^ were applied. Long-range electrostatic interactions were treated by using the particle-mesh Ewald method. The time step used in our MD simulations is 2 fs, and the structures were saved every 100 ps during 0.5 μs of production dynamics with POPS and DOPC/DOPE/DOPS and 0.2 μs production runs with POPC/CHL/PSM and POPE/CHL/PSM. The tetramers were placed with their helix side parallel to the surface on the membrane as a starting orientation for MD runs. Rectangular periodic boundary conditions were applied for all systems in the *xyz*-direction.

To check for self-consistency of MD runs, we further performed repeat MD simulations of a compact helical tetramer on POPS and an extended helical tetramer on DOPC/DOPE/DOPS for 0.2 μs, each starting from a slightly different orientational tilt of the tetramers relative to the membranes. Intermolecular interaction energies (total energy = electrostatics energy + van der Waals energy) between the monomers were used to assess the supramolecular packing of the monomers in the extended tetramers (E_I and E_II) on-membrane (Figure S11). As expected,^47^ the packing is driven by Coulomb electrostatic interactions with only minor contribution from steric van der Waals interactions. The fraction of native contacts, Q,^48^ was computed to estimate the convergence of the MD simulations (Figure S12, see Note S3), and thus the last 100 ns was used for postprocessing and analyses of data for each system. Note that we obtained good convergence during the first 100 ns of MD simulation of tetramers on the mixed membrane systems POPC/CHL/PSM and POPE/CHL/PSM and also for the repeat simulations. Hence, we only performed the extra 0.3 μs extended sampling times for the simulations with the anionic membranes, i.e., POPS and DOPC/DOPE/DOPS.

Well-equilibrated MD simulations were performed with the eight different tetramer-micelle complexes, each simulated for 0.3 μs of simulation time with the same simulation parameters as used for tetramer-membrane simulations. For the extended tetramer, it was observed that the micelle either disintegrates into smaller clusters or one or both micelles leave the site of interaction with the tetramer (Figures S4 and S5). Similarly, the top and side starting configurations of the micelles with the compact tetramer give only weak, if any, interactions (Figure S3). Therefore, we considered the BSC_SDS and BSC_MIXED systems (Figure S2) only for further analyses to directly compare their consistent interactions with the strongly bound micelle on the N-terminal region of the tetramer. Further, to evaluate the differences between the tetramer in its compact and extended conformation, we compared the TSC_SDS and TSE_SDS models.

### Analyses

The conformational energy was estimated to assess the relative thermodynamic stability^3^ of the two designed tetramer polymorphs in bulk water, on membrane surfaces, and in the micelle environments, using the GBMV implicit solvent model generalized Born using molecular volume (MV) implemented in the CHARMM (v40b2) program.^49^

The GBMV module^50^ used the generalized Born method that offers a good approximation of the Poisson–Boltzmann (PB) electrostatic solvation energy calculation with high concordance. It uses highly accurate analytical and grid-based methods to obtain the Born radii with a greater than 0.99 correlation, having the advantage of being generally faster than PB solvers and functional for different force fields. The generalized Born equation for calculating the polar/electrostatic solvation free energy (*G*_pol_) is given by eq 1
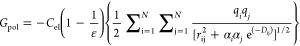
1where *r*_*ij*_ is the distance between atoms *i* and *j*, *q*_*i*_ and *q*_*j*_ are the atomic partial charges, α_*i*_ and α_*j*_ are the effective Born radii of atoms *i* and *j* and depend on the shape of the protein, ε is the (high) dielectric constant of the solvent (water). *D*_*ij*_ is calculated as shown in eq 2
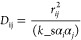
2where *k*_s = 8 for the modified Still’s equation^51^ used here. The conformational energy of the protein tetramers was calculated by isolating the protein structures from the full trajectories containing the protein in the solvated, physiological micelle/membrane environment and then computing the tetramer energy following 200 steps of minimization of each MD snapshot using the GBMV II algorithm.^50,52,53^ GBMV II contains an analytical approximation of the Lee-Richards MV, which avoids high dielectric protein interior solvent-inaccessible regions for computation of nonpolar/apolar free energy of solvation (*G*_apol_), such that the total free energy of solvation (*G*_*sol*_) is given by
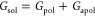
3

Other energy terms, including bonded energy, van der Waals energy, and electrostatic energy, were also calculated with the GB implicit solvent model. The block average method was used to estimate the mean values and standard deviations during the last 100 ns of dynamics, i.e., 1000 statistically independent structures for each system. The interaction energies between the tetramer and membrane/micelles were estimated from the short-range atom-paired Coulombic (electrostatic) and Lennard-Jones (van der Waals) interaction energies in vacuum, computed using GROMACS tools. GROMACS tools was also used to compute hydrogen bond interactions (Figure S7).

## Results

### Preformed Tetramers Interact Weakly with Flat Anionic Lipid Bilayer Membranes

**Figure 1:**
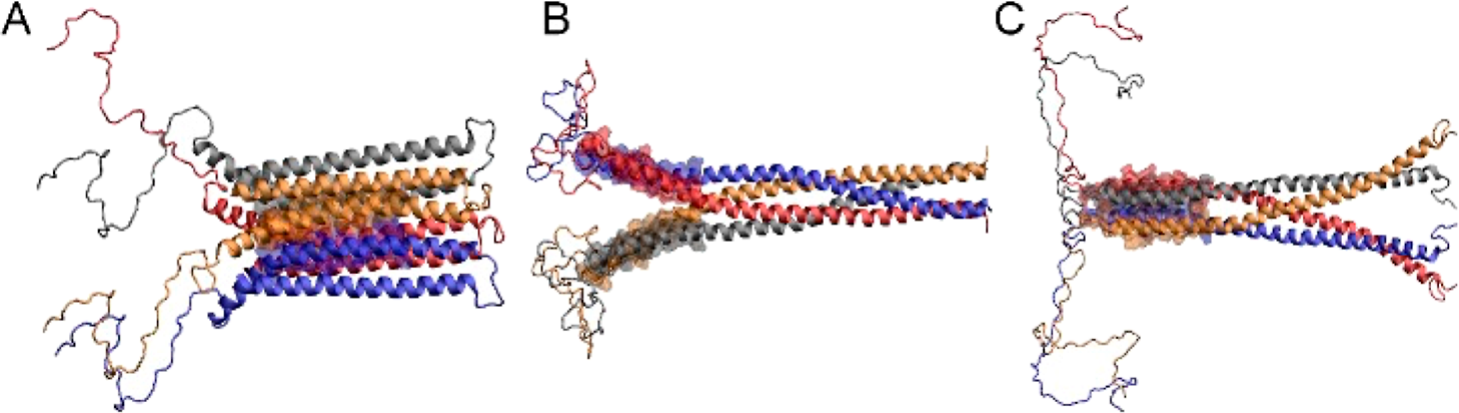
Illustrative conformations of (A) compact conformation of helical αS tetramer, and alternative (B) extended with maximal full-length contacts, and (C) extended with maximized NAC packing. The NAC region (residues 61–95) is highlighted in the overlaid semitransparent surface representation. The snapshots were generated using VMD.^54^

We previously reported that the compact α-helical tetramers and related multimers of αS assemble *via* optimal packing of the hydrophobic nonamyloid-β component (NAC) regions, which are more stable than other molecular arrangements.^26,27^ This design rule was further translated to model two extended 11/3-helical tetramers, Extended I and Extended II models (see Notes S1 and S2 for details of model building and Figure 1 below). Our modeling data on the conformational energies of the systems on membrane surface (Figure S13) and monomer–monomer intermolecular interactions of the extended tetramers reveal that the conformation with maximal full-length contacts was more stable than an alternative with optimized NAC packing. Thus, we focus only on the preferred tetramer with maximal full-length contacts, henceforth referred to as extended tetramer.

**Figure 2:**
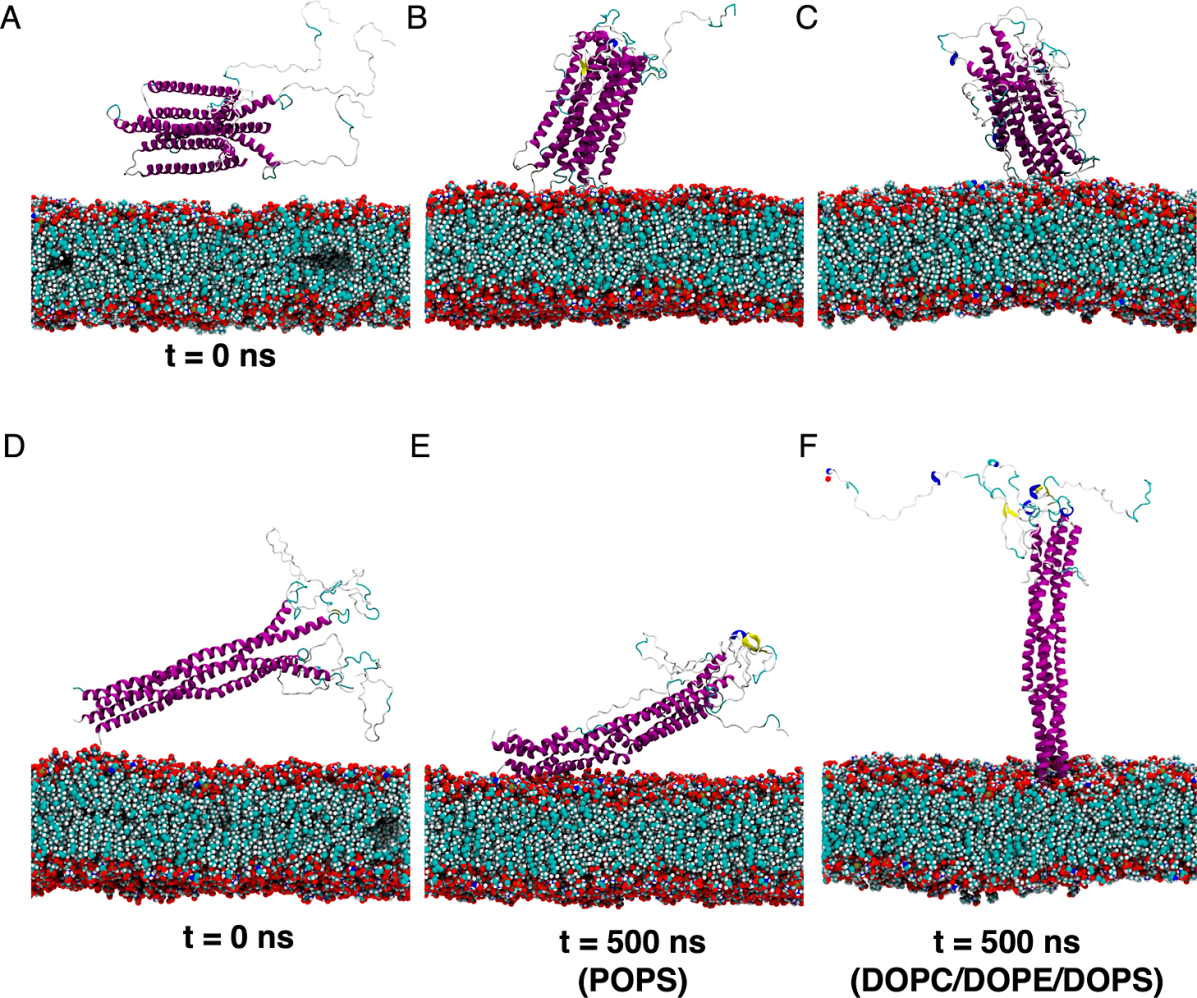
Representative conformations of (A–C) compact and (D–F) extended, helical tetramers adsorbed on anionic membranes POPS and DOPC/DOPE/DOPS. For clarity, water molecules and background ions are not shown. The snapshots were generated using VMD.^54^

The MD models predict that the preformed helical tetramers bind weakly to both anionic membranes, strongly charged POPS, and moderately charged DOPC/DOPE/DOPS. Representative conformations of compact and extended conformations on the membrane are shown in Figure 2.

**Figure 3:**
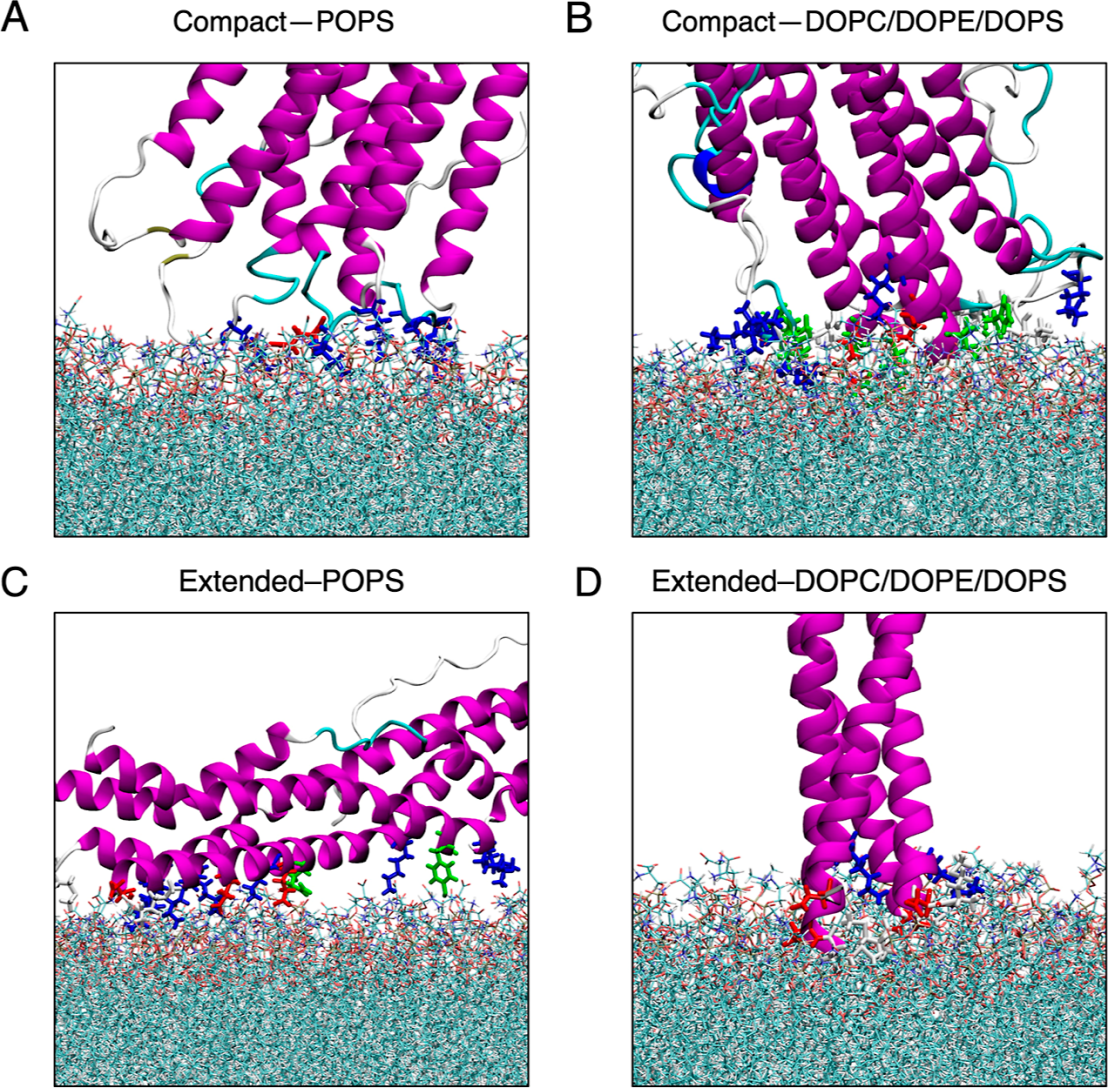
Tetramer residues that are involved in interactions with anionic membranes. (A,B) Interacting residues of the compact helical tetramer with the POPS and DOPC/DOPE/DOPS membrane. (C,D) Interacting residues of the extended helical tetramer with the POPS and DOPC/DOPE/DOPS membrane. Residues are colored by type: basic—blue; acidic—red; polar—green; nonpolar—white. The snapshots were generated using VMD.^54^

A closer view of the residues facilitating the binding to the two different membrane surfaces is shown in Figure 3. Residues in the N-terminal loop region (Lys43–His50) linking two helices of the compact tetramer, in particular, cationic Lys43 and Lys45, stabilize the negatively charged lipid head groups. Note that the tetramer is not significantly deformed at the membrane, and so the neighboring charge-balancing residues (negatively charged Glu46 and Glu57) are also positioned close to the membrane surface. With the decrease of surface negative charge from −1.0*e*/lipid to −0.3*e*/lipid on switching from the POPS to DOPC/DOPE/DOPS bilayer, van der Waals becomes a more obvious secondary interaction, in addition to electrostatic interactions at the αS-membrane interface.

For the compact tetramer, hydrophobic (Val48 and Val49) and polar (His50) residues are found to interact with the membrane. While the compact tetramer displays a similar orientation on both membranes, the extended tetramer adopts different orientations on each. A “lying down” orientation is favored for the extended tetramer on the POPS membrane, whereas the tetramer can sample also a “standing up” orientation on the DOPC/DOPE/DOPS membrane (Figure 2), reminiscent of binding mode orientational selectivity rationally designed for globular proteins *via* strategic placement of polyhistidine-tag anchoring groups.^55^ The binding modes are stabilized by charged interactions with the lipid head groups and subsurface nonpolar interactions with the acyl chain (Figure S14; see Note S3 for a repeat MD simulation).

By contrast, both compact and extended tetramers formed only short-lived, nonspecific interactions on the neutral POPE/CHL/PSM and of POPC/CHL/PSM bilayers (see Figure S12), mostly *via* the disordered C-terminal residues, indicating that the strength of the αS interaction with the lipid bilayers scales with membrane charge. To further probe the influence of surface charge on the interfacial dynamics, we ran duplicate simulations of the compact tetramer on the POPS membrane and extended tetramer on DOPC/DOPE/DOPS with different starting tetramer orientations on the membrane. The repeat dynamics (initially tilted to the membrane) of the compact tetramer adsorbed on the POPS membrane samples show similar orientations as the original trajectory (initially parallel to the membrane), indicating specificity for the compact tetramer structure to interact with strongly charged membranes *via* its loop/kink region. However, the fully upright final orientation starting from a membrane-parallel orientation was not favored for the repeat run when the extended tetramer was initially adsorbed in a tilted orientation on the mixed DOPC/DOPE/DOPS membrane and switched to a more “lying down” orientation similar to that on POPS (Figure 2E). Such dependence on starting configuration highlights the dependence of ergodic short MD runs on initial conditions.

**Figure 4:**
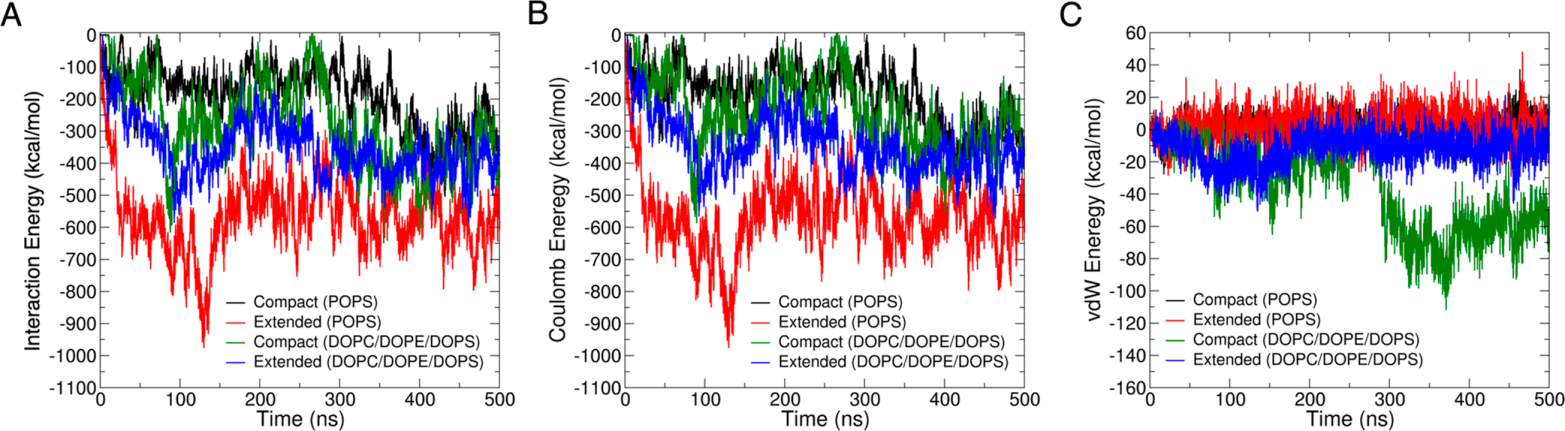
Computed interaction energy between the tetramer and membrane during 500 ns of dynamics with POPS and DOPC/DOPE/DOPS. (A) Total interaction energy; (B) electrostatic interaction energy; and (C) van der Waals (vdW) interaction energy. The interaction energies were calculated using GROMACS tools.

The calculated interaction energies (Figure 4) between the compact and extended tetramers and the two anionic membrane types confirm that electrostatic interactions direct the adsorption of the tetramers on the membranes. Secondary van der Waals interactions contribute ∼10% to binding of the compact tetramer to the DOPC/DOPE/DOPS membrane and are negligible for the other tetramer–membrane complexes. The repeat simulations reflect near-constant interaction strengths for the compact tetramer on POPS (Figure S15), with a slightly stronger complexation energy computed for the extended tetramer in the alternative “lying down” orientation on the mixed membrane bilayer. As reported above, the interactions with different neutral membrane types are nonspecific and short-lived, but predominantly electrostatic in nature, contributing >90% of the total interaction energies.

The difference in total energies observed is related to the mode of interactions. The membranes are near-flat 2D surfaces, which offer only one plane of binding to the tetramer, and the arrangement of the membrane-exposed amino acid residues in the tetramer changes according to the type of preformed helical conformation as noted earlier. The reason for the stronger binding in the extended conformation could be its greater surface area of contact (especially in the “lying down” orientation), which scales according to the overall membrane charge. On the other hand, the exposure of only the loop/kink region in the compact conformation provides fewer contact points with the flat membrane surfaces, resulting in similar sized interaction energies with POPS and DOPC/DOPE/POPS membranes.

### Preformed Tetramers Interact Strongly with Spherical Anionic Micelles

**Figure 5:**
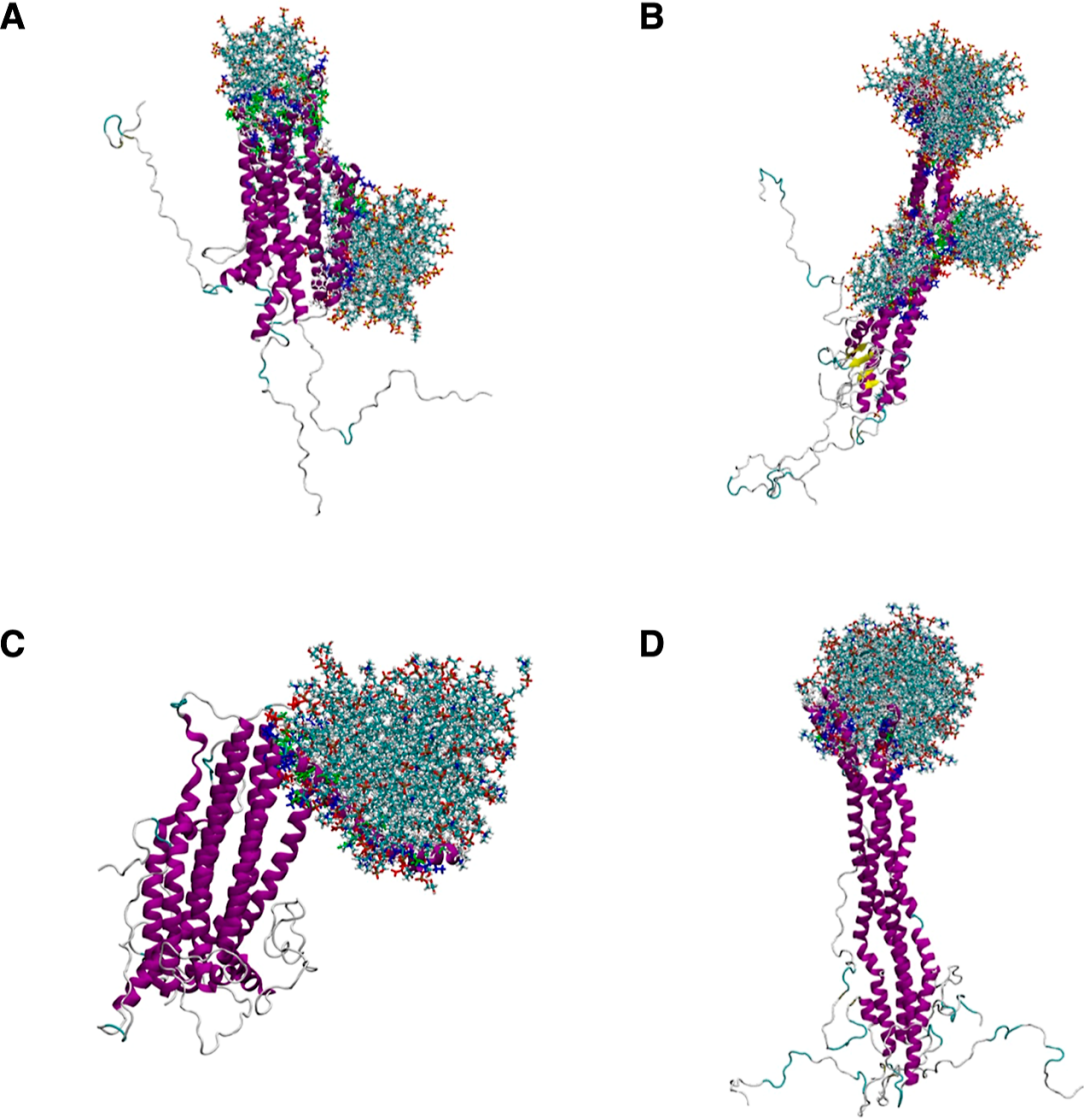
Representative snapshots of the tetramers interacting with micelle nanoparticles. (A) Compact and (B) extended conformations binding SDS micelles and (C) compact and (D) extended conformations binding to mixed micelles. Residues are colored by type: basic—blue; acidic—red; polar—green; nonpolar—white. Note: the micelles were arbitrarily placed near the bottom and side of the tetramers in the starting structures. The snapshots were generated using VMD.^54^

To better understand the predicted conformational preference of αS helical tetramers mediated by weak interactions with flat anionic lipid bilayers, we explored the conformational preferences of both compact and extended tetramers interacting with designed anionic spherical micelle nanoparticles (Figure 5), which are a morphologically different biological surface.

We observe that strongly negatively charged SDS micelles spontaneously disassemble during dynamics, resulting in smaller clusters of micelles making enhanced interactions of SDS with both tetramers. By contrast, the mixed micelles remained intact during MD simulations while interacting with the tetramers, reflecting the reduced intermolecular Coulombic repulsion inside the mixed-composition nanoparticles.

In general, we note that micelle binding displayed regioselectivity, which was not apparent for the flat membranes including micelle-induced small local breaks in the helical continuity. Micelle interactions are primarily observed with residues Lys43 and Lys45 from the N-terminal region of the tetramer, which were also found to interact with the membrane (Figure 3). Additionally, residues Val49 and His50 interacted favorably with the SDS micelle, and Glu46 was found to interact with the GMS2/FOS16/LMPG micelle. Secondary interactions with micelles include the NAC region involving residues Glu62, Val63, Phe66, Phe70, and Val77 for SDS and Glu61, Gln62, Val66, Val70, and Phe94 for GMS2/FOS16/LMPG. The N-terminal loop region and nearby NAC residues drive the interactions with micelles.

**Figure 6:**
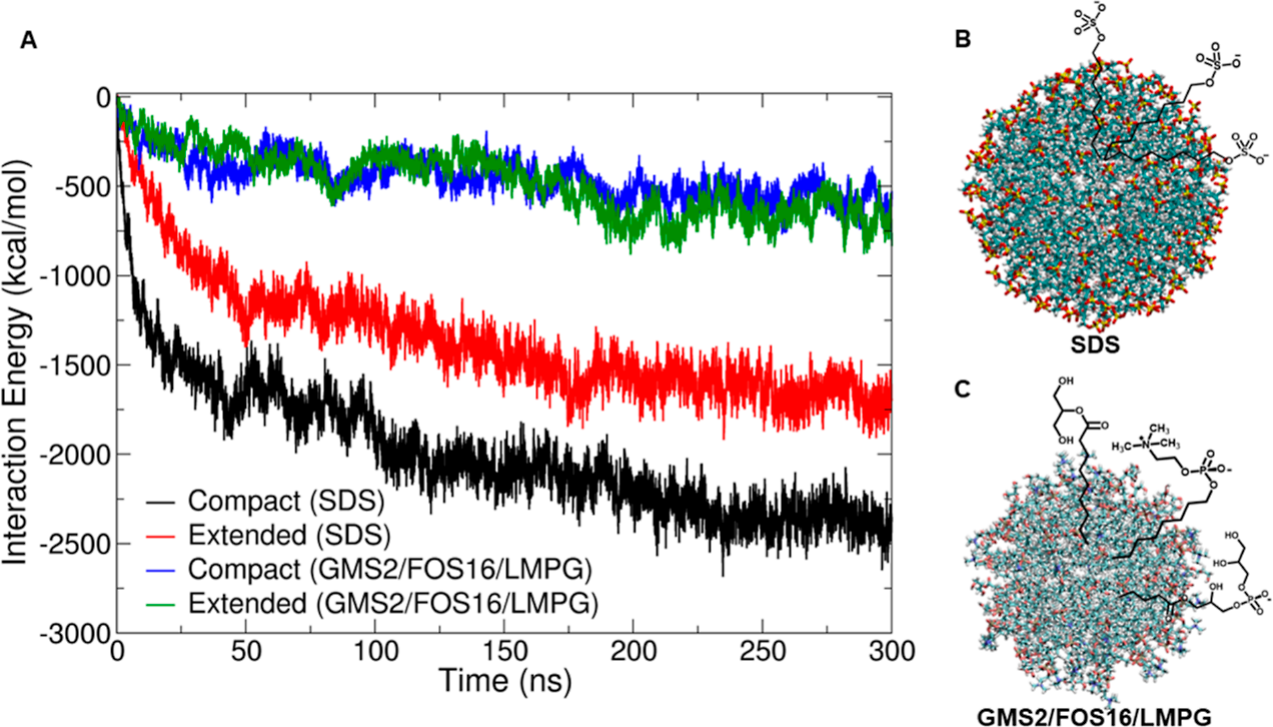
(A) Comparison of timelines of total interaction energies (summed electrostatics and vdW) between the tetramer (in its compact and extended conformations) and each micelle used in this study. Structures of designed spherical micelles with cartoon of the chemical structures: (B) strongly negatively charged SDS [−1.0*e*/molecule] and (C) mildly negatively charged mixed micelle GMS2/FOS16/LMPG [−0.3*e*/molecule]. The snapshots were generated using VMD^54^ and the interaction energies were calculated using GROMACS tools.

Our computed model interaction energies (Figure 6) of the compact and extended tetramers with the strongly negatively charged SDS micelles [-1.0*e*/molecule] and with the moderately negatively charged mixed GMS2/FOS16/LMPG micelles [−0.3*e*/molecule] reveal stronger interactions than with the lipid membrane. This is because the spherical micelle has conformational and translational freedom to sample multiple 3D interaction pockets on the tetramer. With multimicrosecond to millisecond sampling in the future, it is possible that the computed difference in interaction strengths of the tetramer with membrane vs micelle may be narrowed as the tetramer explores more stable conformations, possibly creating local 3D pockets or mini-cavities on the dynamic membrane surface.

In common with the on-membrane simulations, we find mainly electrostatic interactions (Figure S16) and scaling of interaction strengths with lipid charge. The strongly negatively charged SDS micelle facilitates contacts over a wider surface area of the tetramer including the N-terminus bearing the loop region, compared to weaker interactions with the mixed micelle (Figure 6, and see Figures S2–S5), and so we focus on interactions with the SDS micelles only.

**Figure 7:**
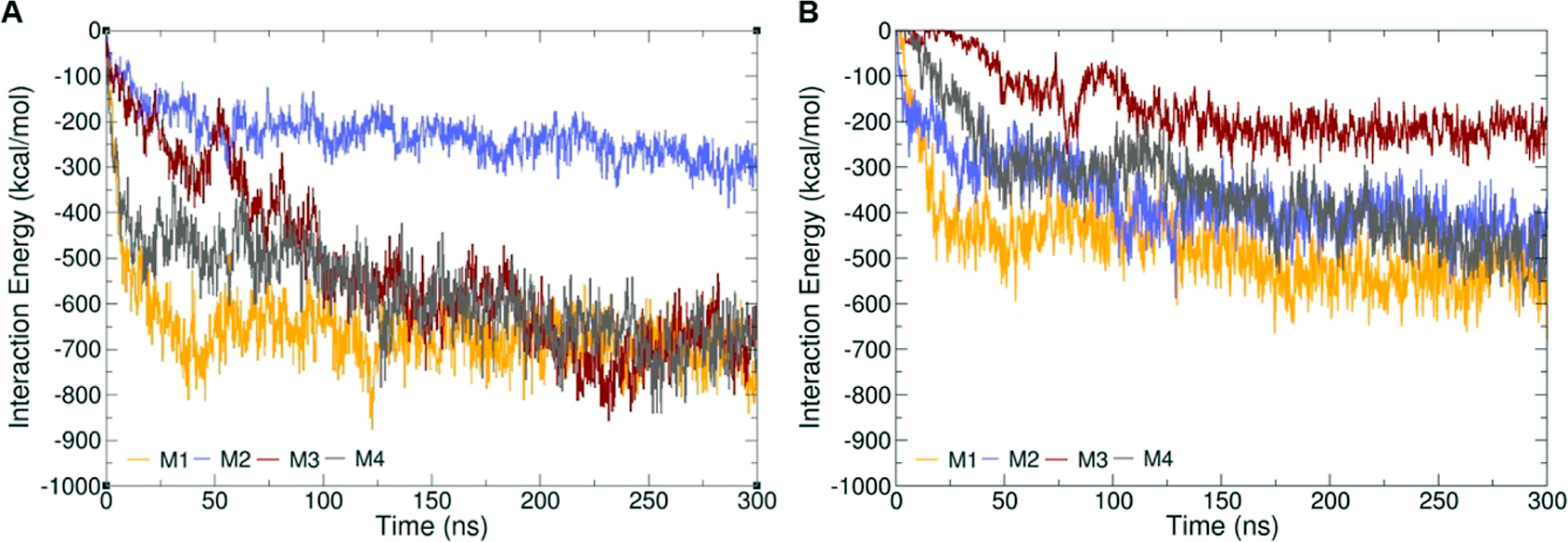
Interaction energy between each monomer (marked M1, M2, M3, and M4) of the tetramer with the micelles in its (A) compact conformation and (B) extended conformation. The interaction energies were calculated using GROMACS tools.

The calculated stronger interaction of the SDS micelles with compact vs extended tetramer structures originates from the difference in supramolecular packing between the monomers within the tetramers in the two different conformations, which changes the presentation of surface-exposed charged and polar amino acids to interact with the micelle, which is driven mainly by electrostatic interactions (Figure 6). We note the small interaction energy difference for compact vs extended tetramers (Figure S17), reflecting the weak interactions of the moderately charged mixed micelle with all regions of the tetramers. On the other hand, the strongly charged SDS micelle shows a much larger difference between the conformations (Figure S17). The interaction strengths reflect the nature of the tetramer–micelle interface, as the strongly negatively charged micelle makes stable, long-lived interactions with the loop and N-terminal regions of the tetramer. For the compact tetramer, three out of four monomers interact strongly with the micelle (Figure 7), while for the extended tetramer, only one out of four monomers interacts strongly with the micelle (see Note S4 for details).

**Figure 8:**
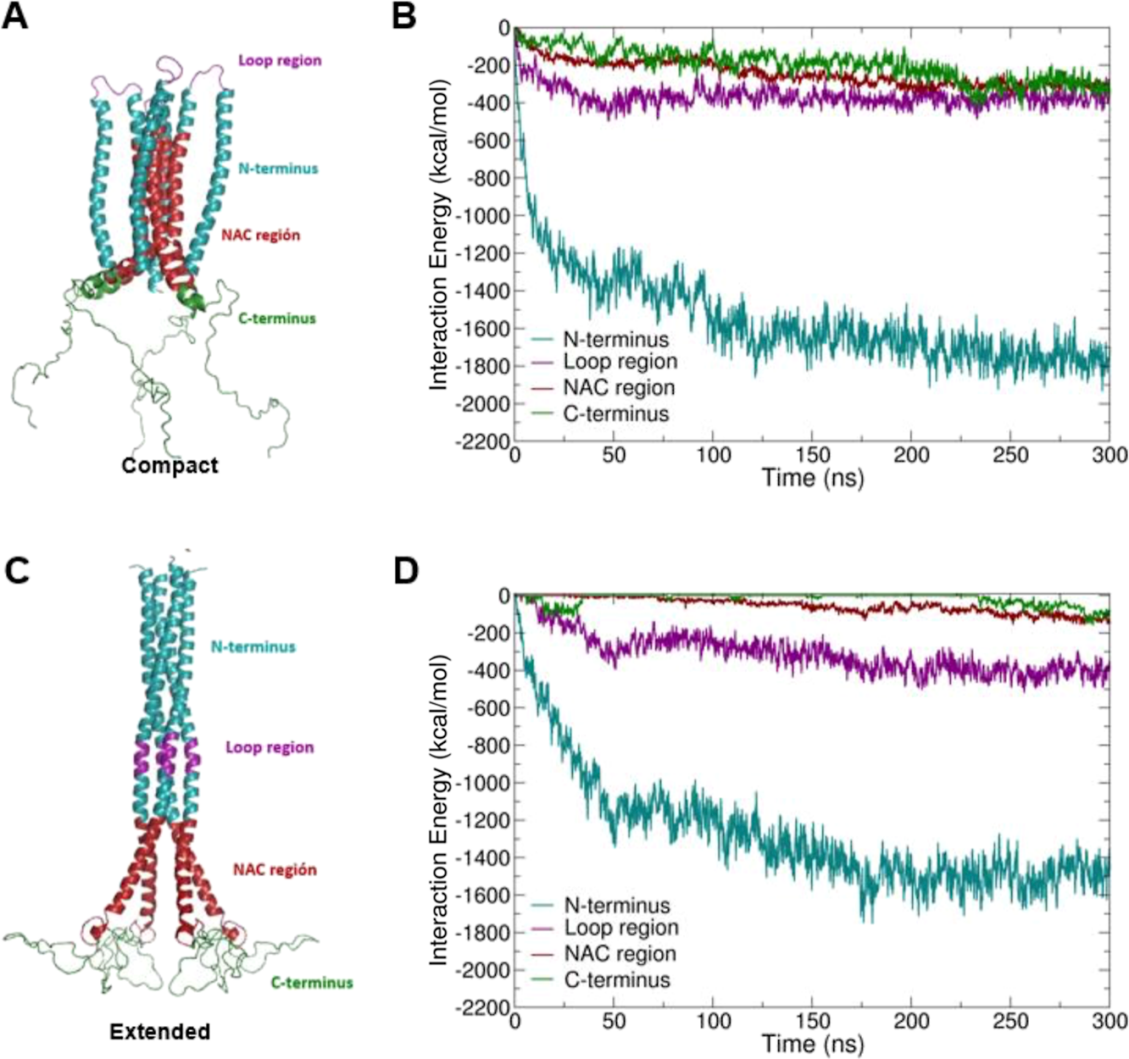
(A) Compact and (C) extended tetramer structures with different topologies: N-terminus, loop region, NAC region, and C-terminus. Interaction energy plots for each region of the (B) compact and (D) extended tetramers with the SDS micelles. The snapshots were generated using VMD^54^ and the interaction energies were calculated using GROMACS tools.

For the extended tetramer conformation (see Figure S6 for convergence of simulation plots), the interactions experienced by each monomer show similar trends, indicating that the symmetry in the tetramer is preserved while interacting with the spherical micelle. This can be seen by comparing the monomer–monomer interaction energy profiles of the tetramers in the presence of the micelle, with respect to their interactions in bulk water without the micelles (Figure S18A,B). Further, we decomposed the interactions based on the distinct regions of the tetramers (Figure 8) such as the N-terminus bearing residues 1 to 60, including the loop region (residues 43 to 50), the hydrophobic NAC region with residues 61 to 95 (which is less exposed in the compact conformation), and the acidic C-terminus with residues 96 to 140. In the compact tetramer, the monomeric C- and N-terminus are more adjacent to each other than they are in the extended conformation, which may facilitate electrostatic interactions with the micelles. In the extended tetramer, the hydrophobic NAC domain is next to the N-terminus, which may partially repel the micelle. Therefore, it would be expected that the compact conformation would interact more strongly with the micelles. This highlights a key point for molecular design and engineering for interactions with biological nanoparticles, in balancing the overall conformation and local topology. Here, the exposed helical surface of the extended tetramer could potentially provide more interaction sites with micelles, but the nature of the exposed amino acids makes it difficult for the extended tetramer to sustain the interactions.

In the compact conformation, the N-terminal region samples the most favorable interactions with SDS, as it is located at the outermost exposed domain of the tetramer. The loop region, which is also part of the N-terminus, contributes ∼25% of the total interactions of the N-terminal region with the micelle. Similar to the compact conformation, the extended conformation interacts most strongly with micelles *via* the N-terminal region.

### Influence of Membrane and Micelle Composition on the Conformational Selection between Compact and Extended Helical Tetramers

**Figure 9:**
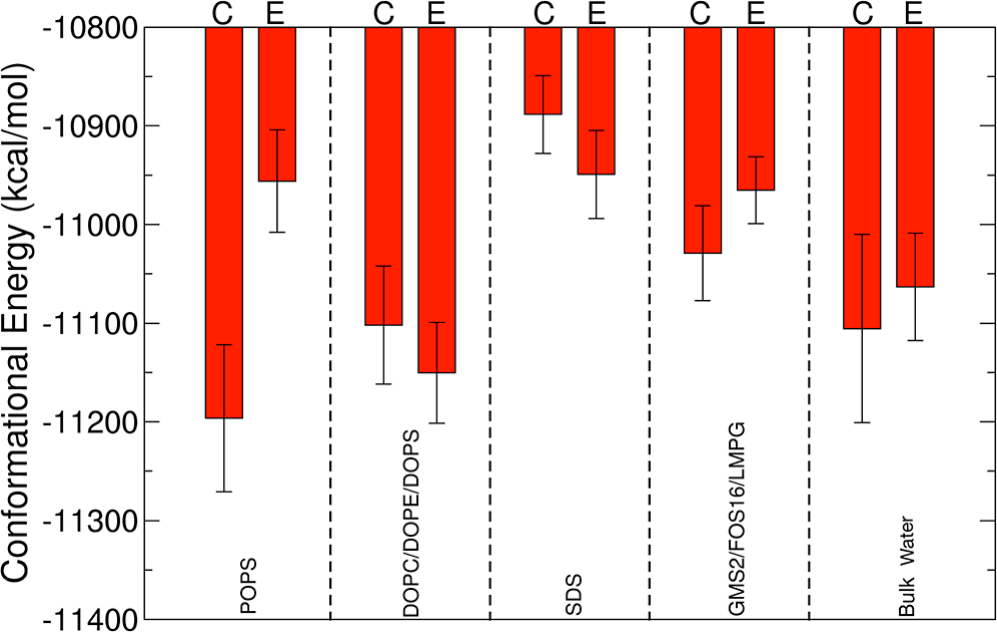
Calculated conformational energy for the compact (denoted as C) and extended (denoted as E) helical tetramers at strongly charged POPS membranes, moderately charged DOPC/DOPE/DOPS membranes, strongly charged SDS micelles and moderately charged GMS2/FOS16/LMPG micelles, and in bulk water solution. The conformational energies were computed using the GBMV II^50,52,53^ method.

Although both types of helical tetramers interact weakly with the model flat lipid bilayer membrane environments investigated here, the conformational energies predict that adsorption on negatively charged biological membranes (POPS and DOPC/DOPE/DOPS) could alter the relative thermodynamic stability of the tetramer polymorphs (Figure 9). In bulk water, the conformational energies predict that the compact tetramer is more stable than the extended tetramer. No significant difference in stability was observed for the compact tetramer between the bulk water and the DOPC/DOPE/DOPS mixed membrane environments resulting in *t*-test *p*-value >0.05. By contrast, improved thermodynamic stability of the extended helical tetramer was found when interacting with the mixed membrane, and significantly altered stabilities of both tetramers when interacting with the single-lipid SDS or POPS membrane, and with all micelles (all *p*-values <0.05). On the neutral membranes, a similar effect was found even in the very weakly membrane-associated states, with stability of the extended tetramer more affected than the compact structure (Figure S19). The above findings suggest that membrane interactions can somewhat improve the thermodynamic stability of the extended helical tetramer and could potentially help impede its degradation or further aggregation. The thermodynamic stabilities of the tetramers as predicted from the GBMV II (see Materials and Methods) conformational energies correlate well with the helical content (Table S3), in line with previous studies of amyloid β (Aβ_13–26_) unfolding.^56^

Crucially, the relative thermodynamic stability of compact vs extended helical tetramer varies with the membrane environment. When associated with the strongly charged POPS membrane, the compact helical tetramer is significantly preferred over the extended helical structure. This is consistent with the high population of compact tetramer conformation that predominates in (charge-rich, high dielectric) water (Figure S19). Upon binding to a moderately charged DOPC/DOPE/DOPS membrane, the preference is reversed, with the extended tetramer predicted to be more stable than the compact tetramer. This suggests that αS tetramers adopt different preferential conformations as they traverse heterogeneous biological environments. The high population of compact tetramer conformation that predominates in solution (Figure 9) could switch to extended conformations on moderately charged membrane surfaces such as DOPC/DOPE/DOPS but revert to compact on either highly charged membranes surface such as POPS or neutral membranes such as POPC/CHL/PSM or POPE/CHL/PSM. We look forward to experimental testing of this predicted effect, which requires development of new experimental protocols beyond the scope of this study.

The overall weak association of αS helical tetramers with the membranes indicates a dynamic partition between membrane-bound and free solution states.^57^ It is well documented that monomeric αS is prone to aggregate into αS oligomers in the absence of membranes or other order-inducing surface or cofactor,^58^ but the solution state may involve another dynamic equilibrium between a soluble helical tetramer and disordered monomers.^59−61^ Control simulations of a completely disordered tetramer on the POPS membrane (Figure S19) showed negligible interactions and contacts between the unstructured tetramer and the membrane, while only a small population (2%) of helical structures was induced upon binding to the membrane. This result suggests that the disordered αS oligomer has negligible affinity toward the membrane as opposed to a fully preformed helical tetramer. The disordered tetramer showed a significantly less favorable conformational energy on the membrane surface, which indicates a strong preference of the membrane for preformed helical tetramers. Finally, we note that a type of αS oligomer in the disordered state was suggested to bind exclusively to the surface of small unilamellar vesicles composed of DOPC/DOPE/DOPS,^24^ suggesting that the variation in the membrane composition may also affect the binding affinity of disordered αS oligomers (in cellular environments that may favor their formation in the first place, e.g., low pH or high temperature). However, it remains difficult to fully model the formation of new helical structures from a completely disordered state of the protein. Advanced sampling atomistic MD simulations at the millisecond time scale performed on high-performance computing platforms may in future enable the required extensive conformational space sampling.^62^ Another issue might originate from the force field employed in our study. We note that CHARMM36m^42^ is benchmarked against both folded and unfolded proteins but further advances in treatment of conformational and nonbonded energies may be necessary to capture slow events such as physiologically relevant folding kinetics and thermodynamics of αS tetramers/multimers.

On the other hand, stronger associations of both compact and extended tetramers with spherical micelles deplete the tetramer conformational energies compared to their energies in bulk water (Figure 9), unlike the on-membrane tetramers. This reduction in thermodynamic stabilities is more prominent when associating with strongly negatively charged SDS micelles, resulting in significant loss of conformational energy of the compact tetramer vs the extended tetramer.

## Discussion

In this section, we further benchmark and discuss our findings by comparison against known experimental behavior. One general finding from experiments is that interactions with heterogeneous biological surfaces can affect the physiological and pathological behavior of αS by altering its conformational plasticity.^63,64^ Our simulation studies confirm that both compact α-helical and extended 11/3-helical tetramers bind weakly to flat anionic lipid bilayers *via* electrostatic interactions between the N-terminus and the lipid head groups and form only sporadic, short-lived interactions with neutral lipid bilayers. We show that association with the moderately negatively charged DOPC/DOPE/DOPS membrane has a negligible effect on the stability of the compact tetramer but significantly improves the thermodynamic stability of the extended tetramer, in line with the experimental finding that αS not only favors an α-helical conformation upon membrane binding but also simultaneously assembles into helical multimers once associated with the membrane.^65^ In NMR studies of αS bound to lipid membranes, three distinct regions (N-terminal, C-terminal, and NAC) were identified to display different roles in the association with membranes, with the N-terminal helical region directly contacting with the membrane.^66^ The proposed interaction model of αS in that study resembled our extended conformation but is less ordered compared with the helical tetramers studied here.

The relative conformational balance between the compact and extended helical tetramers could be shifted by changing the membrane environment from the highly charged POPS to the moderately charged DOPC/DOPE/DOPS to a neutral POPC/CHL/PSM or POPE/CHL/PSM. Specifically, the compact helical tetramer conformation that predominates in the cytosol could undergo weak associations with highly charged and neutral lipid bilayer membranes retaining the compact conformation but could be replaced by a population of extended helical conformations on moderately charged membranes. Thus, the dynamic equilibrium of helical tetramers could vary in different cellular environments depending on the membrane lipid composition. In particular, the dynamic equilibrium between membrane-associated polymorphic helical tetramers could be coupled with the equilibrium between the pathogenic monomer and nonpathogenic helical tetramer in cytosol. For example, the GBA1 (glucocerebrosidase 1) deficiency induces GSL (glycosphingolipid) accumulation, which could destabilize the helical αS tetramer and other multimers in the dopaminergic neurons and promote self-assembly of monomers to toxic αS oligomers.^20^ Moreover, very recent *in vivo* preclinical data indicate that WT GBA1 in PD-like mice improves the tetramer to monomer ratio and αS solubility. The reduced incidence of lipid-associated αS aggregates improves cognitive and motor performance in mice.^67^ Finally, we note that our findings corroborate a recent combined NMR spectroscopy, biophysical, and computational study^68^ that used membrane nanodiscs (NDs) to represent interactions of αS with a membrane. It was proposed that the ND charges could modulate the aggregation dynamics of αS^68^ with a strong aggregation-inhibiting effect in the presence of NDs with 100% anionic lipids.

On the other hand, binding of spherical strongly negatively charged micelles (SDS) produces strong regiospecific interactions *via* the N-terminus and the loop regions of both tetramers, which competitively weakens the supramolecular packing inside the tetramers. Previous studies have reported that low micelle to αS ratios lead to the formation of oligomeric complexes with SDS micelles with relatively limited α-helical content, which may nucleate self-assembly of monomers to form oligomers, protofibrils, and mature amyloid fibrils.^69^ Similarly, a more recent study highlighted that SDS interactions with αS monomer complexes may stimulate the aggregation process due to disruptive hydrophobic interactions between the NAC (nonamyloid beta component) region and the C-terminal.^70^ Our models predict that the designed micelles with spherical amphiphilic structures bearing net charges similar to those of the biological membranes may play a complementary role to that of membranes by screening the aggregation hotspots of aggregation-resistant tetramer morphologies and arresting further toxic self-assembly. A recent study showed that incorporation of Ganglioside micelles into seeded aggregation reactions of amyloid beta (Aβ, implicated in Alzheimer’s disease) slows down the aggregation of the protein *in vivo*.^71^

One of the functional roles of αS involves regulating neurotransmission and synaptic structures as well as maintaining the homeostasis of dopamine in neurons.^72^ The synaptic vesicles contain a high concentration of PC (phosphatidylcholine) and PE (phosphatidylethanolamine) (∼59–78%), moderate levels of PS (phosphatidylserine) (∼12%), and low levels of SM (sphingomyelin) (∼5–7%). Meanwhile, ∼ 40% of the total lipid content is cholesterol.^7^ Such a heterogeneous membrane environment could lead to polymorphic helical αS oligomers with varying region-specific populations, such as the structures computed in the present study of αS adsorbing on POPC/CHL/PSM and POPE/CHL/PSM. In addition to localization in synaptic vesicles, αS also localizes on other membranous organelles, such as the plasma membrane and mitochondria. The outer leaflet of plasma membrane contains a high percentage of PC and SM lipids accounting for most of the raft-like membranes,^73^ but the concentration of PS and PE increases in the inner leaflet of plasma membrane.^7^ The mitochondrial inner membrane has a high concentration of anionic phospholipid of cardiolipin and PE. Based on our findings, the interaction of αS with these diverse biological surfaces could be explored in future modeling-led experiments to further gauge the possibility of using population shifts to reroute the protein assembly.

Note that in addition to the compact α-helical and extended 11/3-helical tetramers studied in this work, membrane-bound αS could also include the compact 11/3-helical monomer and extended regular αS monomer. The former was proposed to consist of either five amphipathic α-helices involving residues 1–93^74^ or two helical regions involving residues 1–41 and 45–94 with a single break at positions 42–44.^75^ The membrane-bound extended helix of αS was proposed from many experimental studies.^23,31,76,77^ Helical tetramers assembled from these monomer conformations could also contribute to the pool of tetramers and should be considered in future efforts to comprehensively map relative populations of helical tetramers across the diverse range of cell membrane compositions that are present *in vivo*.

## Conclusions

To summarize, in the present work, we modeled the aggregation-impeding, preformed αS tetramers and found that the relative thermodynamic stability of the compact and the extended helical tetramers could be shifted by the variation of heterogeneous surface charge through weak associations with the membrane and strong associations with the micelles. The predicted propensity of micelle nanoparticles to influence the thermodynamic stabilities of αS helical tetramers points toward the targeted delivery of artificially designed external micelles to impede further αS aggregation from preformed tetramers. Future experiments and simulations could further explore how variation in the biological surface environment mediates αS aggregation at different stages, producing polymorphic species of varying solubility and stickiness that influence the assembly and aggregation paths from monomers to oligomers to fibrils.

Mapping the interaction of αS with 2D membranes and 3D micelles revealed significant shifts in the binding landscape of αS helical tetramers, where the data reveal that the interactions of both compact and extended tetramers are driven by the overall charge and shape of the binding surface, which in turn drives changes in the conformational selection of the different αS structures, providing a potential means of rerouting protein self-assembly and aggregation pathways.

Our simulations reveal that the polymorphic conformations of helical αS tetramers interact weakly with different lipid bilayers *via* weak electrostatic attractions, where the membranes act as a support surface that generally improves their helical stabilities on-membrane. On the other hand, the tetramers interact *via* strong, regioselective electrostatic attractions with negatively charged micelles, remaining bound to the tetramer for the full duration of 300 ns dynamics, screening tetramer sites including the aggregation “hot spots” to potentially inhibit further aggregation.

Our most important new physical insight is that the conformational preference and further assembly of helical tetramers are dependent on cellular conditions and surface environment, but both conformational selection and further assembly occur through the same regions and selectivity criteria, which may explain why it remains challenging to crystallize αS tetramers. Shifts in population balance from aggregation-prone disordered monomers to aggregation immune folded helical tetramers in the cytosol or from helical monomers to helical tetramers on membrane could further stabilize aggregation resistant helical tetramer and help suppress aggregation into alternative, neurotoxic oligomers.

Based on the modeling data and comparison with experiments to-date, we propose a therapeutic design strategy for PD where soluble nontoxic αS tetramers could in the future be repopulated and stabilized by exploiting conformational selection at diverse cellular biological surfaces and synthetic structures such as lipid cubic phase formulations^78^ and other future nanoengineered materials.^79,80^ While such future work is beyond the scope of the present work, our modeling data show that modulating of membrane charge may be one way to drive equilibrium dynamics in αS species and conformations. Stronger interactions with micelles could screen aggregation hot spots to impede or slow further aggregation, which can be considered a second way to disrupt the aggregation process, providing insights into future design rules for new therapeutic alternatives. Future studies could more comprehensively scan the extensive sampling space of the hard-to-detect polymorphic αS tetrameric species to further assess the impact of adsorption on biological surfaces. In particular, the dynamic equilibrium between tetramers and disordered αS monomers may be predictable using advanced sampling methods, such as replica exchange MD.^62^ Such nonequilibrium methods could help remove any conformational bias in the tetramer folding landscape due to the chosen starting configurations, a potential limitation of the current study. Beyond PD, preservation of the helical structure is of broad interest as a building block for biological and bioinspired self-assembled materials, with helix–helix interactions driving efficient supramolecular assembly of natural and designed protein nanostructures.^81−85^

## Data Availability

The GROMACS 2018.4 software used in this work to produce the molecular dynamics trajectories is free and open-source software. Freeware VMD 1.9.3 and XMGrace programmes were used for visualization and plotting, respectively. All membrane-tetramer and micelle-tetramer models were developed in our computational study with their dynamic self-consistency ascertained from the cross-correlation of predicted properties. All force field parameters used were standard CHARMM force field parameters as referenced in the text. All GROMACS input files and model coordinates used in this study are available through the open repository: 10.5281/zenodo.13309803.

## Supporting Information Available

Notes include extended helical tetramer models; effect of monomer packing on thermodynamic stabilities of extended 11/3 helical tetramers; repeat MD simulations; monomer-monomer interactions in tetramer-micelle complexes; convergence of MD simulations; and intermonomer hydrogen bonds in tetramers in the presence of micelles; figures include structural alignment of helical monomer with tetrabachion; interactions of compact tetramer and extended tetramer conformations with highly and moderately charged mixed micelles; computed total, electrostatistics, and vdW interaction energies between tetramers and micelles; timelines of number of hydrogen bonds and of conservation of secondary structure of tetramers; different initial orientations of compact and extended helical tetramers; MD simulations of tetramers; change in distance between COM of tetramer and membrane; comparison of total tetramer-membrane interaction energies of repeat simulations; total monomer-monomer interaction energies; RMSD and fraction of native contacts of tetramer conformation; and conformation and interaction energies, number of contacts and snapshots of disordered tetramer of POPS membrane; tables include calculated conformation energy for the compact and extended helical tetramers in the presence of different neutral membranes; details of tetramer-micelle complex systems; and summary of averaged helix percentage and conformational energies of the system (PDF)

## Abbreviations

PD: Parkinson’s disease
αS: α-synuclein
POPS: palmitoyl oleoylphosphatidyl serine
DOPC: dipalmitoylphosphatidyl choline
DOPE: dipalmitoylphosphatidyl ethanolamine
DOPS: dipalmitoylphosphatidyl serine
POPC: palmitoyl oleoylphosphatidyl choline
POPE: palmitoyl oleoylphosphatidyl ethanolamine
CHL: cholesterol
PSM: palmitoyl sphingo myelin
SDS: sodium dodecyl sulfate
GMS2: glycerol monostearate 2-isomer
FOS16: *N*-tridecyl phospho choline
LMPG: 2,3-dilauroyl-d-*glycero*-1-phosphatidyl-glycerol
