# Supplementary material for: Conformational Selection of α-Synuclein Tetramers at Biological Interfaces
Source: J Chem Inf Model. 2024 Oct 8;64(20):8010–23. doi: 10.1021/acs.jcim.4c01459 (PMC11523075; doi:10.1021/acs.jcim.4c01459)
Supplement: Supplementary file 1 — ci4c01459_si_001.pdf [file ci4c01459_si_001.pdf]

Supporting Information

On the Conformational Selection of α-Synuclein Tetramers at Biological Interfaces

Shayon Bhattacharya[§,†], Liang Xu[§,†], Lily Arrué[§,†], Tim Bartels[¥] and Damien Thompson[§,]*

[§]Department of Physics, Bernal Institute, University of Limerick, V94 T9PX, Ireland

[¥]UK Dementia Research Institute, University College London, WC1E6BT, United Kingdom

[†]These authors contributed equally to this work. *E-mail: Damien.Thompson@ul.ie

Index **Pages**

**Supporting Notes**	**S1.** Extended helical tetramer models.<br>**S2.** Effect of monomer packing on the **thermodynamic** stabilities of extended 11/3-helical tetramers.<br>**S3.** Repeat MD simulations.<br>**S4.** Monomer-monomer interactions in tetramer-micelle complexes.<br>**S5.** Convergence of MD simulations.<br>**S6.** Intermonomer hydrogen bonds in tetramers in presence of micelles.	**S4**<br>**S5**<br>**S6**<br>**S7**<br>**S7**<br>**S8**
**Fig. S1**	Structural alignment of extended helical monomer with tetrabrachion, models of extended helical tetramers and their thermodynamic stabilities. Selected models of full-length extended helical tetramers before and after dynamics and comparison of extended tetramers with compact tetramer	**S9**
**Fig. S2**	Interactions of compact tetramer conformations with highly charged micelles and moderately charged mixed micelles placed initially at the bottom and the side of the tetramer constructs.	**S10**
**Fig. S3**	Interactions of compact tetramer conformations with highly charged micelles and moderately charged mixed micelles placed initially at the top and the side of the tetramers.	**S11**
**Fig. S4**	Interactions of extended tetramer with highly charged micelles and moderately charged mixed micelles placed initially at the bottom and the side of the tetramers.	**S12**
**Fig. S5**	Interactions of extended tetramer with highly charged micelles and moderately charged mixed micelles placed initially at the top and the side of the tetramers.	**S13**
**Fig. S6**	Computed total, electrostatics and vdW interaction energies between the tetramers and the micelles with the micelles placed initially at the top and the side, and at the bottom and the side of the tetramer constructs.	**S14**
**Fig. S7**	Timelines of number of hydrogen bonds (#H-bonds) for compact and extended system.	**S15**
**Fig. S8**	Timelines of conservation of the secondary structure (%) of the tetramer with the designed SDS micelles.	**S16**
**Fig. S9**	Timelines of conservation of the secondary structure (%) of the tetramer with the designed mixed micelles.	**S17**
**Fig. S10**	Timelines of conservation of the secondary structure (%) of the isolated tetramer in bulk water.	**S18**
**Fig. S11**	The intermolecular interaction energy between monomers within each extended tetramer.	**S19**
**Fig. S12**	Two different initial orientations of compact and extended helical tetramers for MD runs on the POPC and mixed DOPC/DOPE/DOPS membrane surfaces. Representative binding conformations of compact and extended helical tetramers interacting with the neutral lipid bilayers POPC/CHL/PSM and POPE/CHL/PSM. The fraction of native contacts Q for all αS tetramers.	**S20**
**Fig. S13**	MD simulations of extended tetramers interacting with two types of anionic membranes.	**S21**
**Fig. S14**	The change in the distance between the COM of tetramer and membrane. The initial minimum distance between tetramer and membrane is 15 Å and 5 Å. The number of contacts between the	**S22**

	tetramer and membrane surfaces. The simulation box of the extended αS tetramer with the DOPC/DOPE/DOPS membrane.	
**Fig. S15**	Comparison of the total tetramer–membrane interaction energies of repeat simulations. Computed interaction energy between the tetramer and the membrane with neutral membrane types POPC/CHL/PSM and POPE/CHL/PSM.	**S23**
**Fig. S16**	Total monomer-monomer interaction energies of both tetramer conformations in bulk water.	**S24**
**Fig. S17**	Plot of the difference for Interaction Energies for both conformations compact and extended interacting with the strong negatively charged SDS micelle (black line) and with the moderately charged micelle GMS2/FOS16/LMPG (green line).	**S25**
**Fig. S18**	Root Mean Squared Deviation and fraction of native contacts (Q) of tetramer conformation.	**S26**
**Fig. S19**	Conformational energy, number of contacts, interaction energy, and snapshots disordered tetramer on POPS membrane.	**S27**
**Table S1**	Calculated conformation energy for the compact and extended helical tetramers in the presence of different neutral membranes. MD simulations of an unstructured αS tetramer on the POPS membrane.	**S28**
**Table S2**	Details of the tetramer-micelle complex systems.	**S28**
**Table S3**	Summary of the helix percentage averaged over the last 100-ns trajectory of each system.	**S29**
**Table S4**	Summary of conformational energies and helical percentages of two extended and one compact helical tetramer in water.	**S29**
**Supporting References**		**S30**

Supporting Notes

S1. Extended helical tetramer models. The experimentally determined 11/3-extended helical models of lipid-bound αS (Ser9 – Ala89) monomers were kindly provided by Prof. Ralf Langen as obtained from the study by Jao *et al* [1]. From an ensemble of ten extended helical monomer conformations, we selected the monomer model with the least root mean square deviation (RMSD) from a monomer of tetrabrachion computed from TM-align [2] server (see **Figs. S1A**, **B**), a naturally occurring right-handed coiled coil symmetric tetramer (PDB code 1FE6 [3]). From the extended helical monomer model selected, the extended helical tetramer models were then constructed.

We designed four tetramer (residues 9–89) complex variants utilizing the curvature geometry of 11/3 helices (**Fig. S1C**): (I) *Separated and optimised* in which the hydrophobic core of NAC (71 – 82) is optimized for intermolecular contact between hydrophobic residues of monomers, while the rest of the monomer helices are separated from each other, (II) *Intertwined and optimised* in which the hydrophobic core contacts are optimized but the rest of the monomer helices are intertwined with each other, (III) *Intertwined and not optimised* in which the core contacts are not optimized but focus is given on intertwining the helices so that it may form maximum contacts in other regions. This could be taken as a control to see if the hydrophobic residues play a role in stabilizing the extended helical tetramers as well, and (IV) *Aligned and optimised* in which the hydrophobic core of NAC region of each monomer was aligned to each other to optimise the tetramer structure containing a C_4 symmetry axis. All complexes were generated preserving the original curvature of the selected monomer model with the Lys residue sidechains facing outwards.

To account for the thermodynamic stabilities of the full-length tetramer models for model selection, we did a preliminary test on the helical stabilities of the NAC region (E61 – A89) by running short molecular dynamics (MD) simulations for 50 ns (**Fig. S1D**, **E**). Models I and IV present very similar topologies in the NAC region with the optimized hydrophobic core contacts (as also predicted from our previous compact helical tetramer models [4]). However, the NAC helices are more stable in model IV than I. Thus, we did not consider model I for further analyses. On the other hand, the initial NAC helical topology in model II is also mostly preserved after 50 ns, while for model III (not optimized with core

hydrophobic contacts), the NAC helices come closer to each other during the 50 ns dynamics. To account for the impact of the C- and/or the N-terminus (especially the intertwined N-terminal regions in models II and III) on the overall inter-helical hydrophobic contacts and thermodynamic stabilities, we chose models II, III and IV to further design full-length extended helical tetramer αS (1 – 140) models by adding the N- and the C-terminus to αS (9 – 89).

To have a stable extended helical tetramer model as a starting conformation to further study their adsorption on cellular membrane surfaces, we constructed the full-length αS models II, III and IV (**Fig. S1F**). The tetramer structures after 100 ns of MD simulations in aqueous solution (**Fig. S1G**) reveal that model II loses the extended structural integrity, while models III and IV retain their structures. The total conformational energies (**Table S1**) translate to their overall stabilities which follows the order, III > IV > II, with model III being most stable and model II the least stable. The differences are majorly contributed from the electrostatic energies, which for models II and III are appreciably higher than model IV, meaning more charge-charge repulsions in II and III. However, this is highly compensated in III by their most favorable vdW energy. Thus, we discard model II from further analysis and pick models III and IV which we name as Extended I and Extended II, respectively.

We also accounted for the prevalence of extended and compact tetramers in solution (**Fig. S1H**). The conformational energies (**Table S4**) reveal that compact αS helical tetramer is more stable in water than the extended helical tetramer with maximum helicity retained. The energy barriers show that it is easier to transit from Extended I to Compact than from Extended II to Compact.

S2. Effect of monomer packing on the thermodynamic stabilities of extended 11/3-helical tetramers

Our previous studies report that by optimising the helical packing of the hydrophobic non-amyloid-β component (NAC) regions, the compact α-helical tetramers and related multimers of αS assemble to stable constructs [4, 5]. To test the applicability of this design rule to the extended 11/3-helical tetramers, we constructed two distinct extended helical tetramer models (Extended I and Extended II models, see **Methods**). Extended I display maximal full-length contacts and Extended II shows maximal hydrophobic contacts in the

NAC regions (see **Fig. 1**). As a result, the NAC region close to the C-terminus of Extended I is more accessible to solvent due to the helical curvature, while the N-terminal region of Extended II is more exposed. We performed 500 ns MD simulations of each tetramer on two negatively charged membranes (POPS and DOPC/DOPE/DOPS, see Methods). Details of model selection, repeat MD simulations and convergence are given in **Figs. S1 – S4**.

The calculated conformational energy shows that the Extended I tetramer is more stable than the Extended II analogue when interacting with both types of membranes (**Fig. S13A**). The net stability of Extended I over Extended II model is contributed from the inter-monomer vdW energies (**Fig. S13**). This finding suggests that close packing of helix-helix contacts in the Extended I model stabilizes the αS tetramer structure and that surface interactions *via* the N-terminal region could stabilize the extended 11/3-helical tetramer. Representative conformations of Extended II tetramer on two different negatively charged membranes are shown in **Fig. S13B**.

Given the calculated conformational energies, we discounted Extended II from further analyses and focus only on the more preferred Extended I tetramer (henceforth referred to as extended tetramer, unless otherwise specified) in the following discussion. From now on we will refer to the tetramer in its two conformations: Compact **(Fig. 1A)** and Extended **(Fig. 1 B)** to analyse its main modes and zones of interaction with different charged particles and how its stability can be affected by such interactions.

S3. Repeat MD simulations. We ran duplicate simulations of compact tetramer on the POPS membrane surface and extended tetramer on the DOPC/DOPE/DOPS membrane starting from a different tilted orientation to the membrane (**Fig. S12**; see **Methods**). The repeat dynamics (initially tilted) of compact tetramer adsorbed on the POPS membrane adopts similar orientation as the original dynamics (initially parallel) emphasising rigidity in the compact tetramer structure when interacting with negatively charged membranes (**Figs. S12A**,**B**) *via* their loop/kink region. However, the "standing up" orientation was not sustained for the repeat run of extended tetramer adsorbed on DOPC/DOPE/DOPS membrane, and they adopt a "lying down" orientation (**Fig. S12B**) more like their POPS-bound conformation (**Fig. S12E**). This reflects the propensity of the mixed DOPC/DOPE/DOPS bilayers to sample non-uniform contact points with the tetramer due to uneven charge distribution on

their surface leading to a greater conformational freedom than their POPS-associated counterpart, more prominent for the extended tetramer.

S4. Monomer-monomer interactions in tetramer-micelle complexes. The timelines of interaction energy profiles of compact tetramer-micelle suggest interactions with regards to M2 is least favourable, since the micelle is interacting with other monomers (M1, M3 and M4), leaving M2 isolated. The interactions with micelle improve over time for M3 and M4 considerably since it could be observed that the monomer-micelle interactions become more favourable until 300 ns, while the M2 samples most favourable and stable interactions with the SDS micelle from the beginning. This is because the M1 is first approached by the micelle, before interacting with M4 and M3, while M2 remains the least interacting monomer isolated (**Fig 7**).

As seen with the compact tetramer system, in the extended conformation it is observed that one of the monomers, M3 is not interacting as much with the micelle, while M1 is interacting most as it has the most favourable interaction energy (**Fig. 7**). M2 and M4 interact favourably with the micelle but to a much lesser extent than observed for other monomers. The major contribution in monomer-micelle interactions come from the Coulomb electrostatics. monomer-monomer interactions in

S5. Convergence of MD simulations. The time evolution of the fraction of native contact Q [6] was calculated to estimate the convergence of the present MD simulations (**Fig. S12D–G**). Different effects of membrane composition on the same compact or extended tetramers were observed. The more highly charged POPS membrane seems to better maintain the starting tightly packed NAC conformation of compact tetramer than the mixed DOPC/DOPE/DOPOS membrane. By contrast, a higher Q was maintained for the extended tetramer when interacting with the mixed DOPC/DOPE/DOPS membrane. Such results further support the hypothesis that the relative stability of pre-formed helical αS tetramers, and population shifts between different tetramer conformers, are sensitive to the membrane composition. The fraction of native contacts was calculated according to the formula:

$$Q(X) = \frac{1}{N}\sum_{(i,j)} \frac{1}{1+\exp\left[\beta\left(r_{ij}(X)-\lambda r_{ij}^{0}\right)\right]} \qquad \text{(S1)}$$

Heavy atoms *i* and *j* in residues θ_i and θ_j are in contact if the distance between them is less than 5.0 Å. r_{ij} (X) is the distance between *i* and *j* in conformation X; r^0_{ij} (X) is the distance in the native state (starting conformation). β is a smoothing parameter taken to be 5 $Å^{-1}$ and the factor λ accounts for fluctuations when the contact is formed, taken to be 1.8 for the all-atom model.

S6. Intermonomer hydrogen bonds in tetramers in presence of micelles. The timelines of hydrogen bonding shows that both tetramers present almost the same amount of intermonomer hydrogen bonds in the presence or absence of the micelle, with a slight deficit while interacting with the SDS (**Fig. S7**). This is because the monomer-monomer packing is lost to some extent, which is consistent with the timelines of conformational energy and the fraction of native contacts (**Fig. S6**). It is possible that protein-protein H-bonds also count helical H-bonds within a single tetramer monomer, and thus similarities with and without SDS are seen.

Supporting Figures

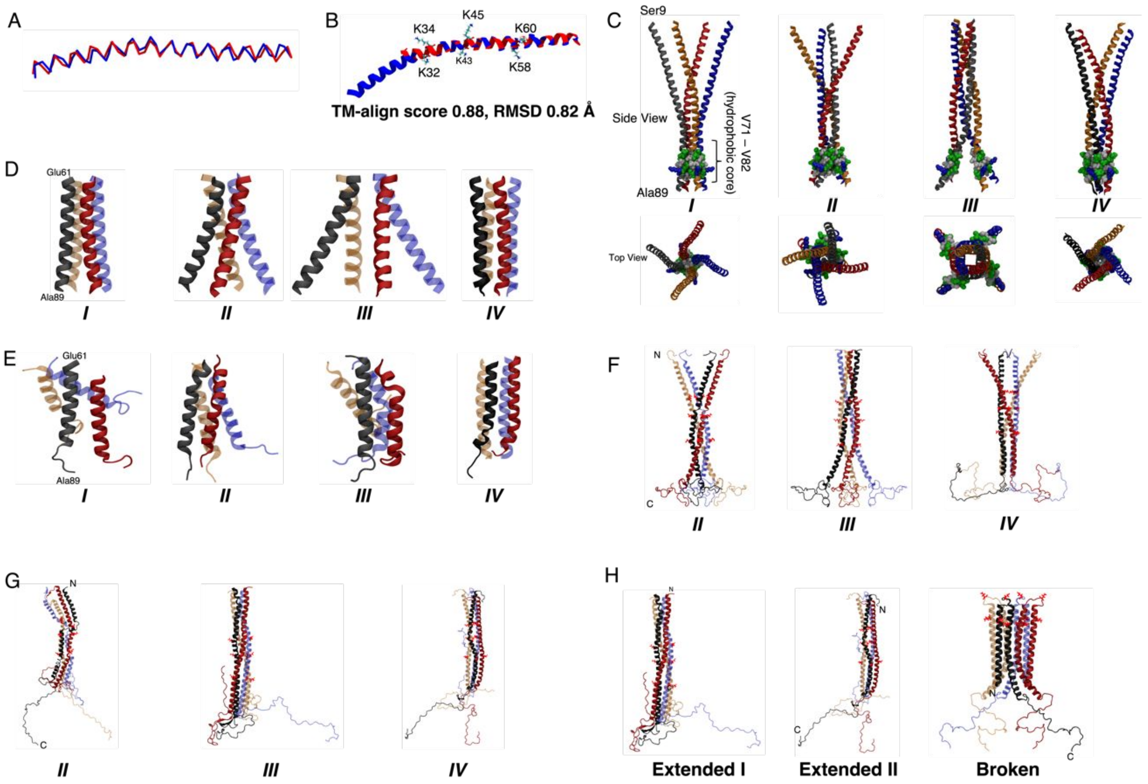

Figure S1. (A) Overlay of Cα atom traces of αS (blue) with the coiled-coil monomer of tetrabrachion (red, PDB 1FE6 [3]). (B) Structural alignments of the αS (9 – 89; blue) extended helical model (out of 10) with tetrabrachion (red) with TM-align [2] score which gives the least RMSD (0.82 Å). The Lysine side chains (K32, K34, K43, K45, K58, and K60) can be seen perpendicular to the helical axis and facing outwards to facilitate interaction with membrane. Tetramer models were further built based on this model αS (S9 – A89). (C) Structures of four (I – IV) designed extended helical tetramer models of αS (9 – 89) from side and top views. The hydrophobic core of NAC is shown as spheres. (D) Initial structures of four αS (61 – 89) NAC helical tetramer models. (E) Final structures of the four NAC tetramer models after 50 ns of dynamics. (F) Starting structures (side and top views) of full-length αS (1– 140) selected extended helical models II, III and IV. (G) Extended helical structures of selected models after 100 ns free dynamics in aqueous solution. (H) Shortlisted models (two extended and one compact) for studying adsorption of tetramers on cellular membrane surfaces. Models III and IV in panel B are renamed as Extended I and II, respectively.

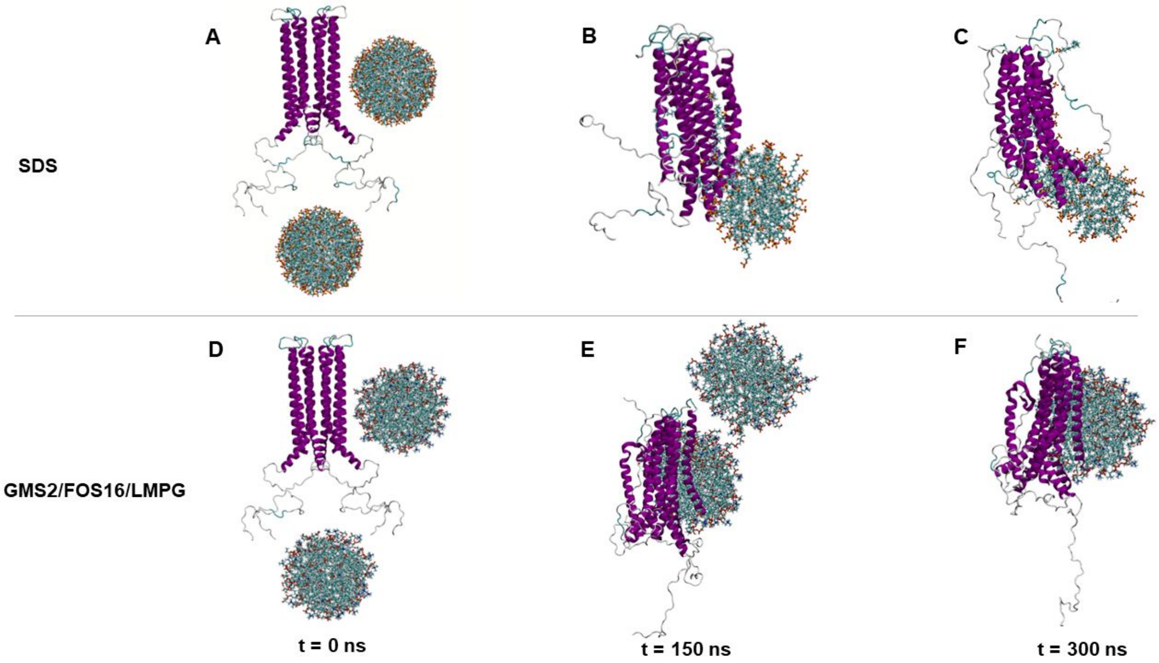

Figure S2. Interactions of compact tetramer conformations with (A, B, C) highly charged (SDS) micelles and (D, E, F) moderately charged (GMS2/FOS16/LMPG) mixed micelles placed initially at the bottom and the side of the tetramer constructs. Tetramer-micelle conformations after (B, E) 150 ns and (C, F) 300 ns of dynamics are shown. It is observed that the micelle initially at the bottom of the tetramer leaves contact with the tetramer while the micelle placed in the side interact with the N-terminal region of the tetramer.

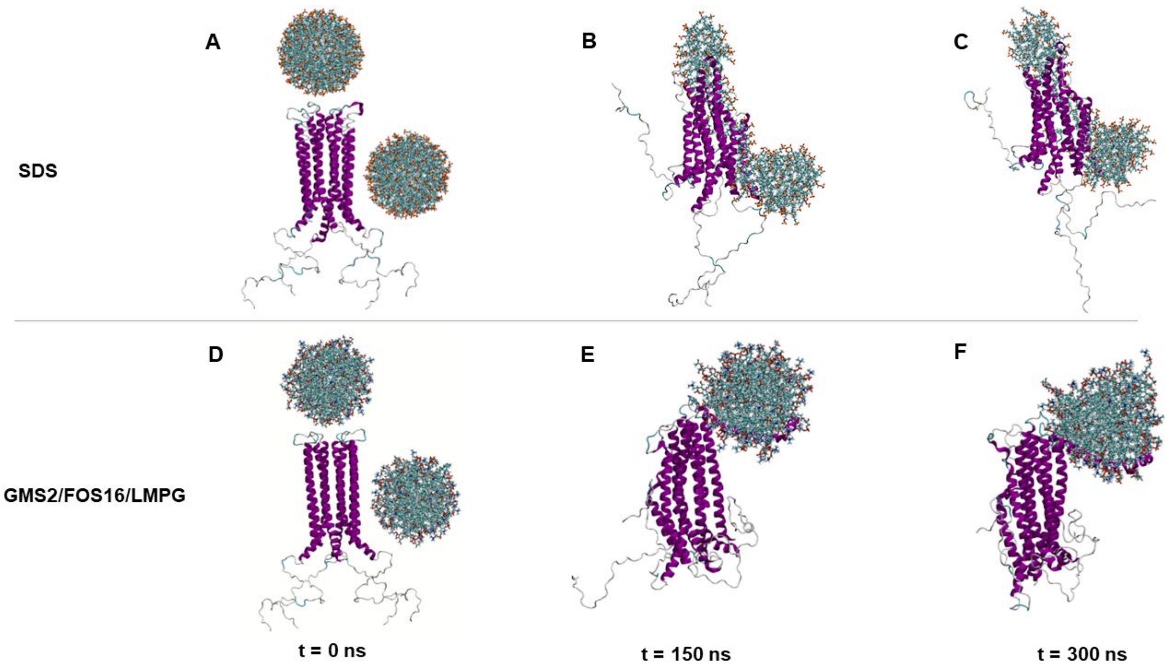

Figure S3. Interactions of compact tetramer conformations with (A, B, C) highly charged (SDS) micelles and (D, E, F) moderately charged (GMS2/FOS16/LMPG) mixed micelles placed initially at the top and the side of the tetramers. Tetramer-micelle conformations after (B, E) 150 ns and (C, F) 300 ns of simulations are shown. With both SDS micelles, the interactions mainly localise in the loop and N-terminal region of tetramer (B, C). However, with the mixed micelle, there is not as much affinity towards the tetramer due to the lower charge of the micelle, and interactions are limited to the side (N-terminal region) of one of the monomers, with no interacting loop region.

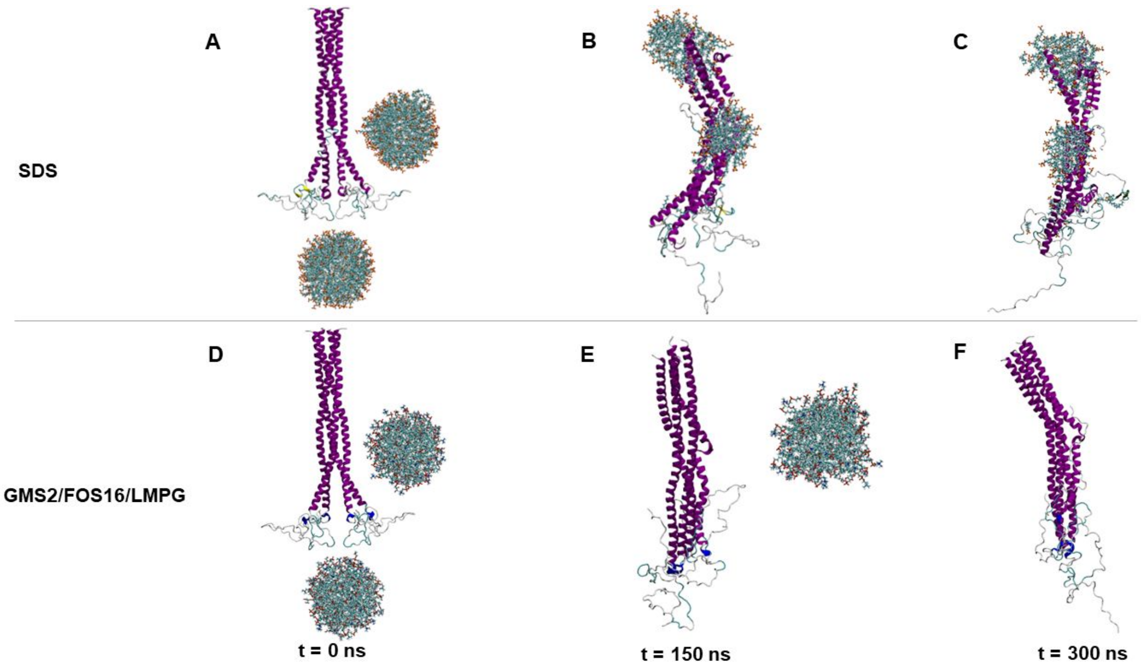

Figure S4. Interactions of extended tetramer with (A, B, C) highly charged (SDS) micelles and (D, E, F) moderately charged (GMS2/FOS16/LMPG) mixed micelles placed initially at the bottom and the side of the tetramers. Tetramer-micelle conformations after (B, E) 150 ns and (C, F) 300 ns of dynamics are shown. Similar to the interactions with the compact tetramer, it is observed that the bottom micelle in both cases has no interactions and moves away from the tetramer. However, the SDS micelle placed at the side of the tetramer separates into smaller clusters, while interacting with the loop and the N-terminal region. The moderately charged mixed micelle does not have strong interactions eventually moving away from the extended tetramer.

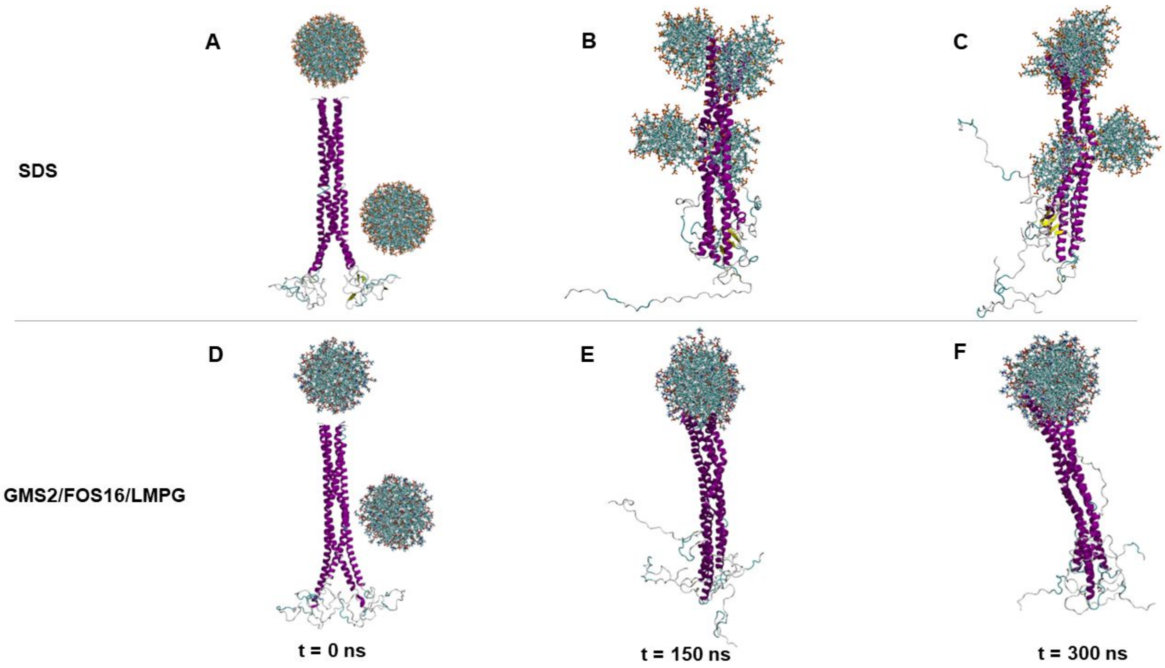

Figure S5. Interactions of extended tetramer with (A, B, C) highly charged (SDS) micelles and (D, E, F) moderately charged (GMS2/FOS16/LMPG) mixed micelles placed initially at the top and the side of the tetramers. Tetramer-micelle conformations after (B, E) 150 ns and (C, F) 300 ns of dynamics are shown. Here again the SDS micelles presents interactions with both N-terminal region and the loop region, just like the compact tetramer with SDS system. With regards to the mixed micelles, one of the micelles only present a slight affinity for the N-terminal region of the extended tetramer, much like the compact tetramer interacting with the mixed micelles.

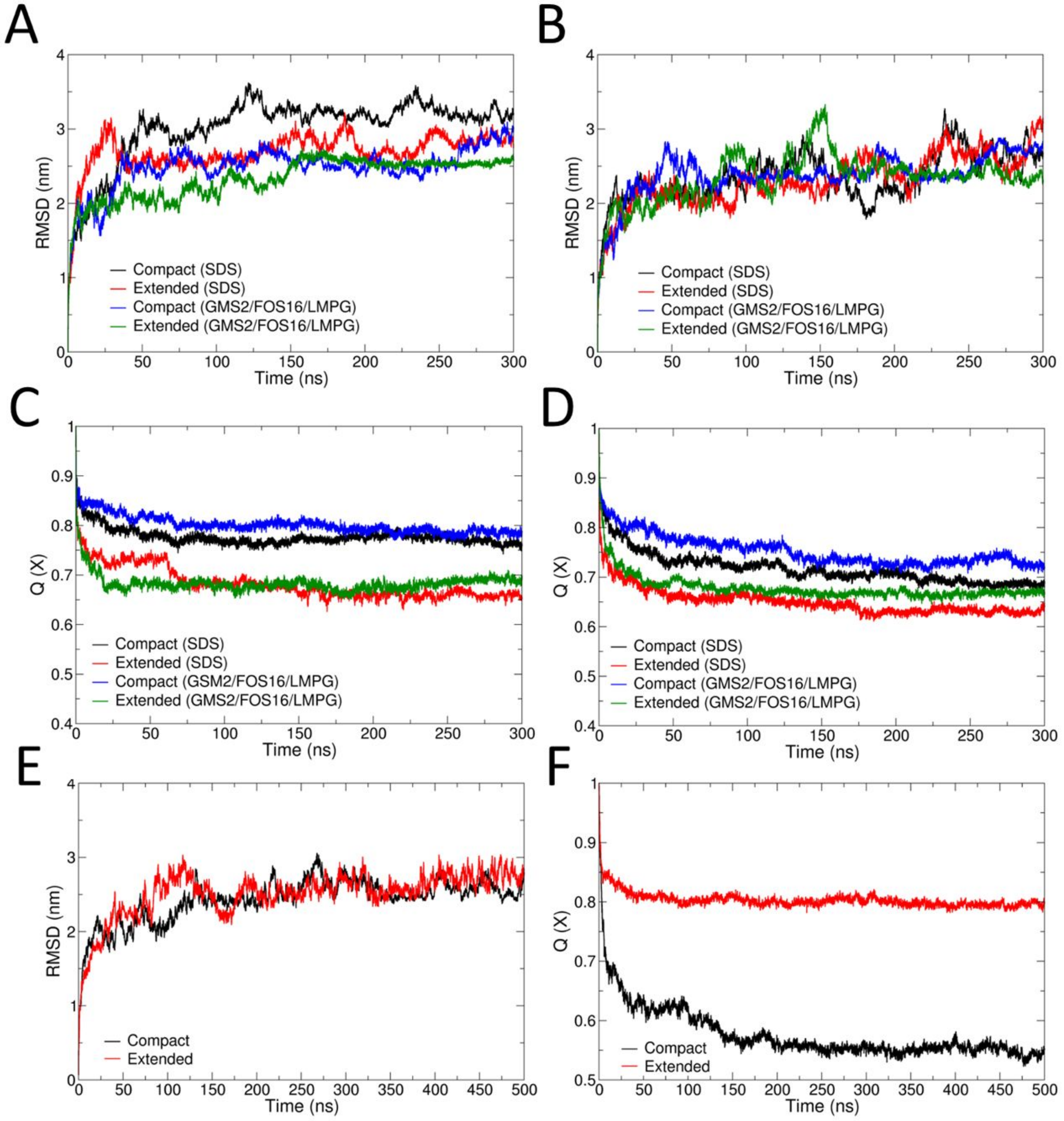

Figure S6. Root Mean Squared Deviation (RMSD) and fraction of native contacts (Q) of (A, C) tetramer conformation with two micelles positioned initially on the bottom and the side of the tetramer, (B, D) tetramer conformation with micelles positioned initially on the top and the side of the tetramer, and (E, F) isolated tetramers without micelle in bulk water.

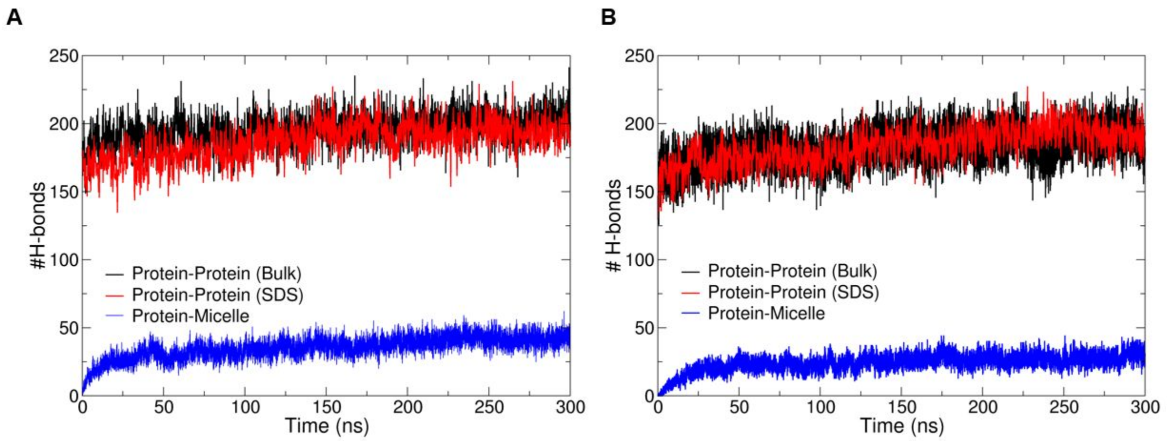

Figure S7. Timelines of number of hydrogen bonds (#H-bonds) for compact (A) and extended system (B), where the black line corresponds to the hydrogen bonds within the tetramer, in bulk. The red line corresponds to the hydrogen bonds within the tetramer, while interacting with SDS. And the blue line corresponds to the hydrogen bonds between the tetramer and the micelle.

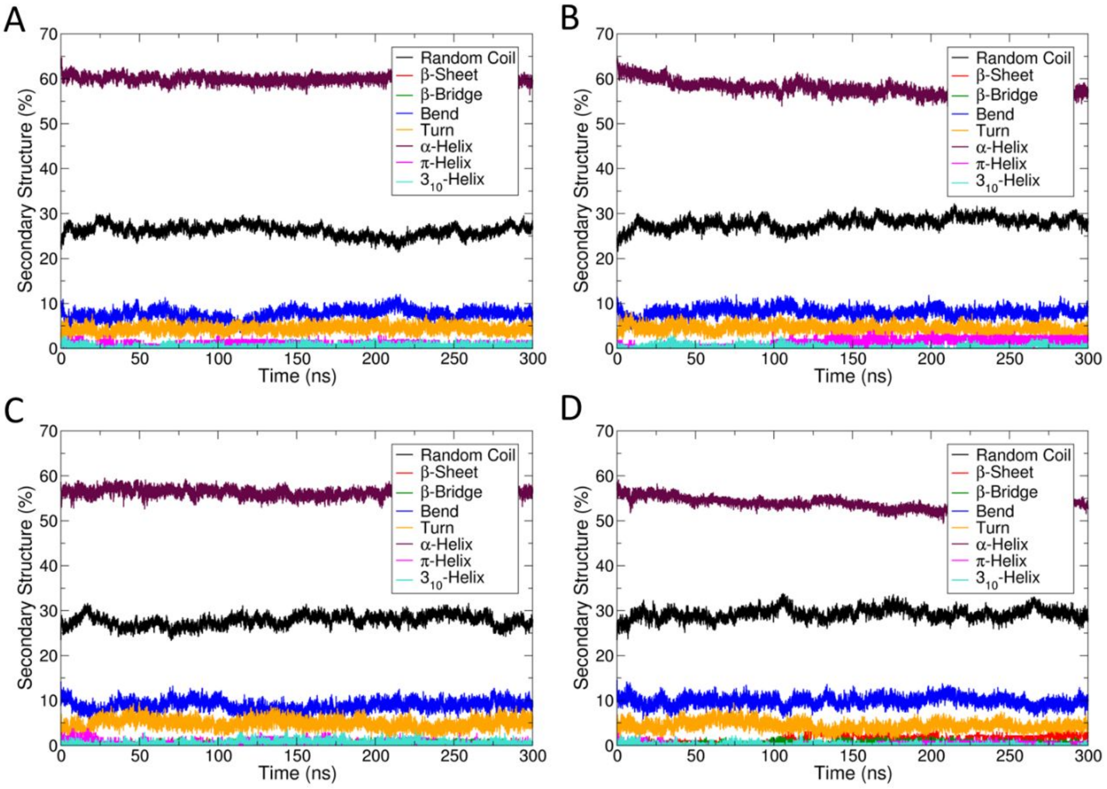

Figure S8. Timelines of conservation of the secondary structure (%) of the tetramer with the designed SDS micelles. (A, C) Compact conformation with starting micelle conformations on the bottom and the side. (B, D) Extended conformation with starting micelle conformations on the top and side.

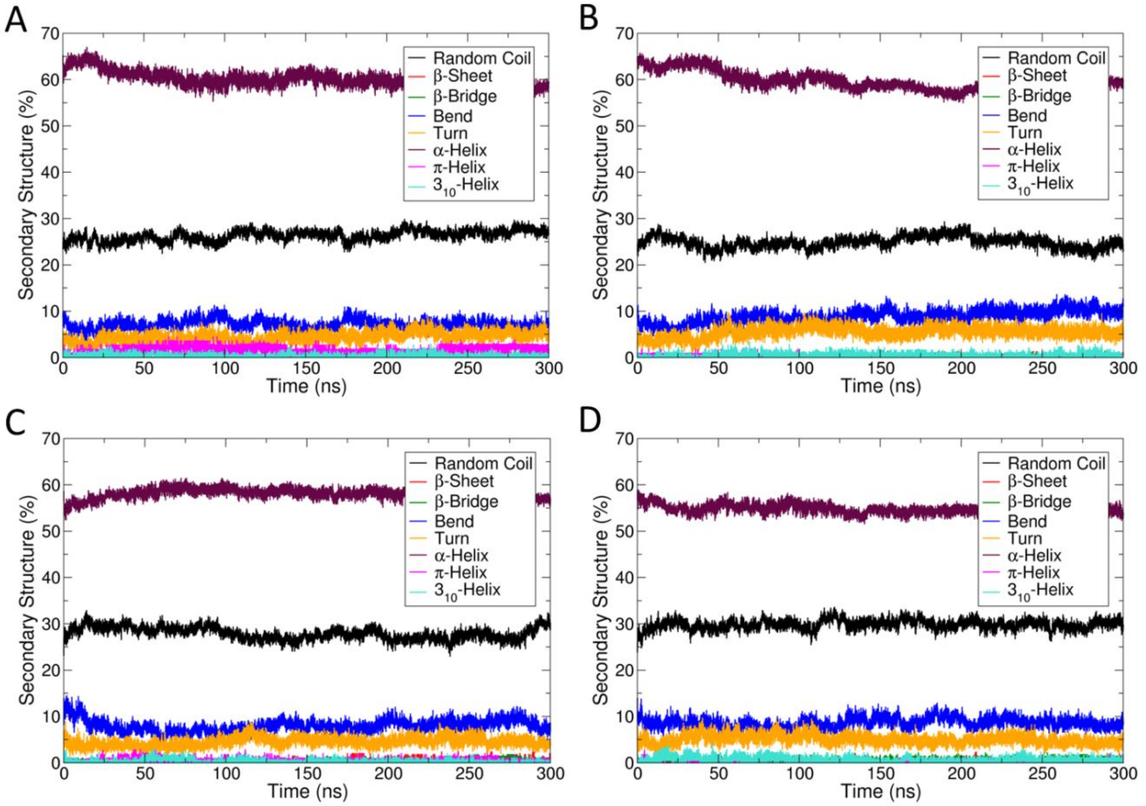

Figure S9. Timelines of conservation of the secondary structure (%) of the tetramer with the designed GMS2/FOS16/LMPG micelles. (A, C) Compact conformation with starting micelle conformations on the bottom and the side. (B, D) Extended conformation with starting micelle conformations on the top and the side.

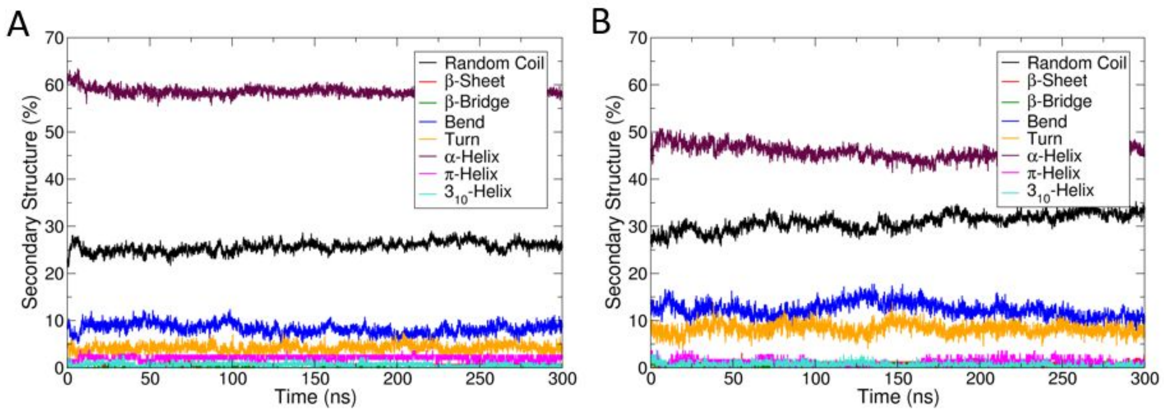

Figure S10. Timelines of conservation of the secondary structure (%) of the isolated (A) compact and the (B) extended tetramer conformations in bulk water without micelles.

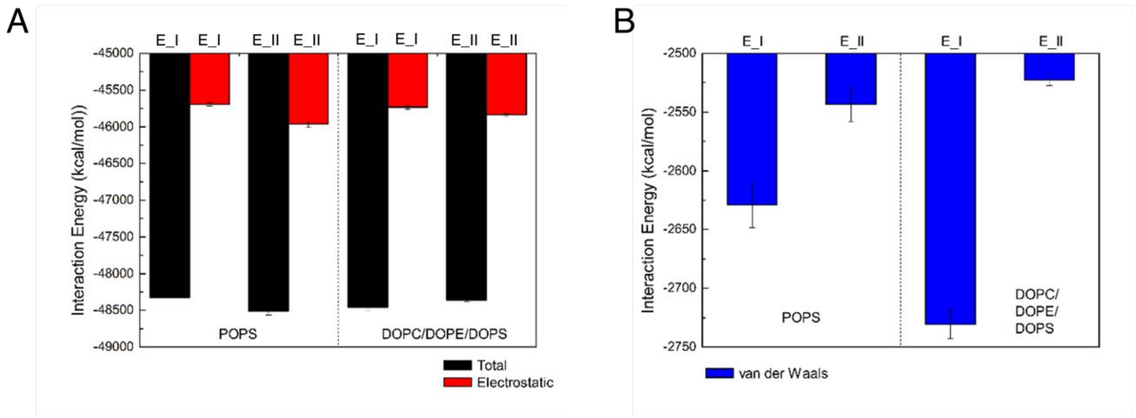

Figure S11. The intermolecular interaction energy showing (A) total energy and Coulombic electrostatic energy, and (B) vdW energy between monomers within each extended tetramer (E_I and E_II) based on the last 100 ns trajectory of each system (tetramer on membrane) in bulk water.

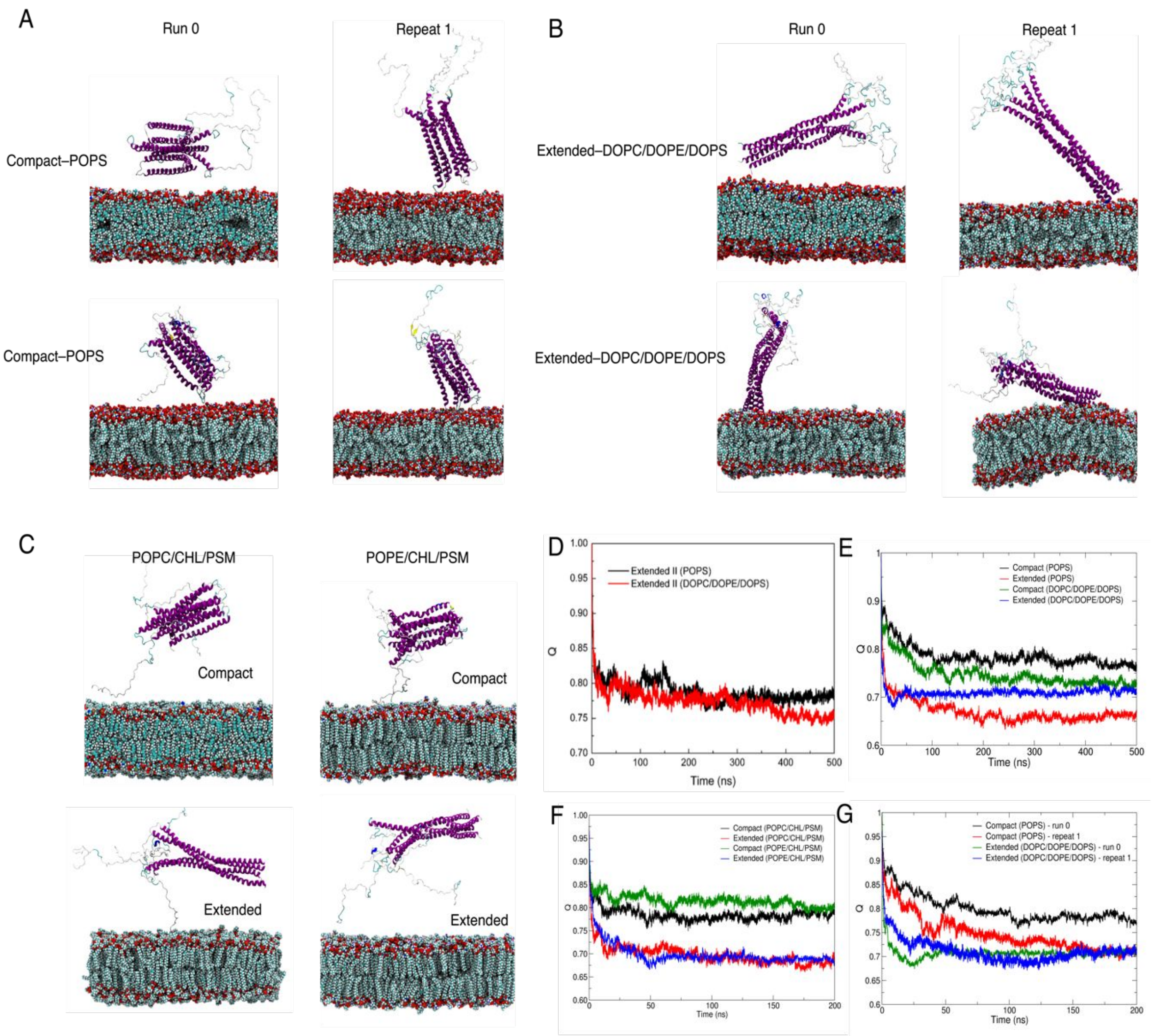

Figure S12. Two different (Run 0 = initially parallel to membrane and Repeat 1 = initially tilted to membrane) initial orientations of compact (A) and extended (B) helical tetramers were chosen for MD runs on the POPC and mixed DOPC/DOPE/DOPS bilayer membrane surfaces. The representative binding conformations are shown in lower panels. (C) Representative binding conformations of compact and extended helical tetramers interacting with the ternary mixture of lipid bilayers POPC/CHL/PSM and POPE/CHL/PSM. For clarity, water molecules and background ions are not shown. (D–G) The fraction of native contacts Q for all αS tetramers.

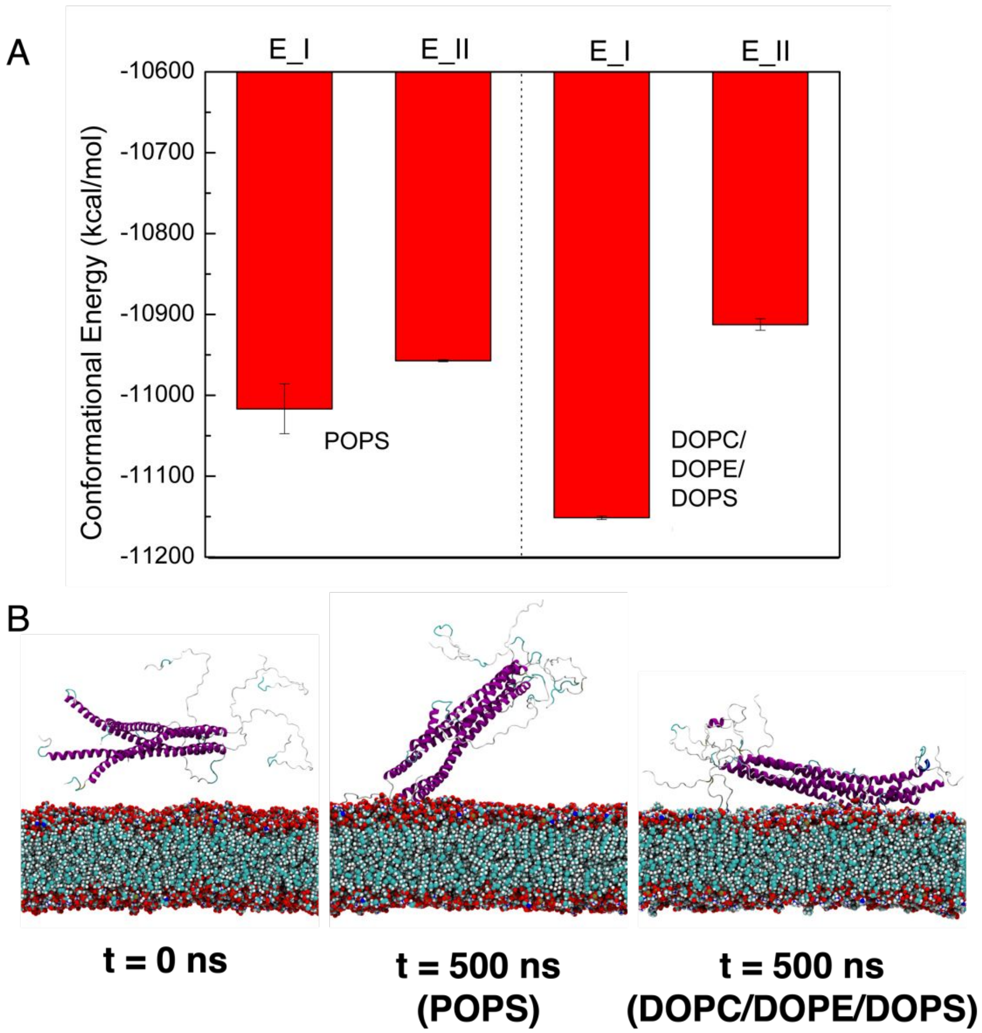

Figure S13. MD simulations of extended tetramers interacting with two types of anionic membranes, starting from an initial protein–surface separation of 5 Å. (A) Comparison of the calculated conformational energies for the extended helical tetramers interacting with different types of membranes. E_I and E_II refer to the Extended I and Extended II models, respectively. (B) Representative conformations of approaching and membrane bound Extended II tetramer.

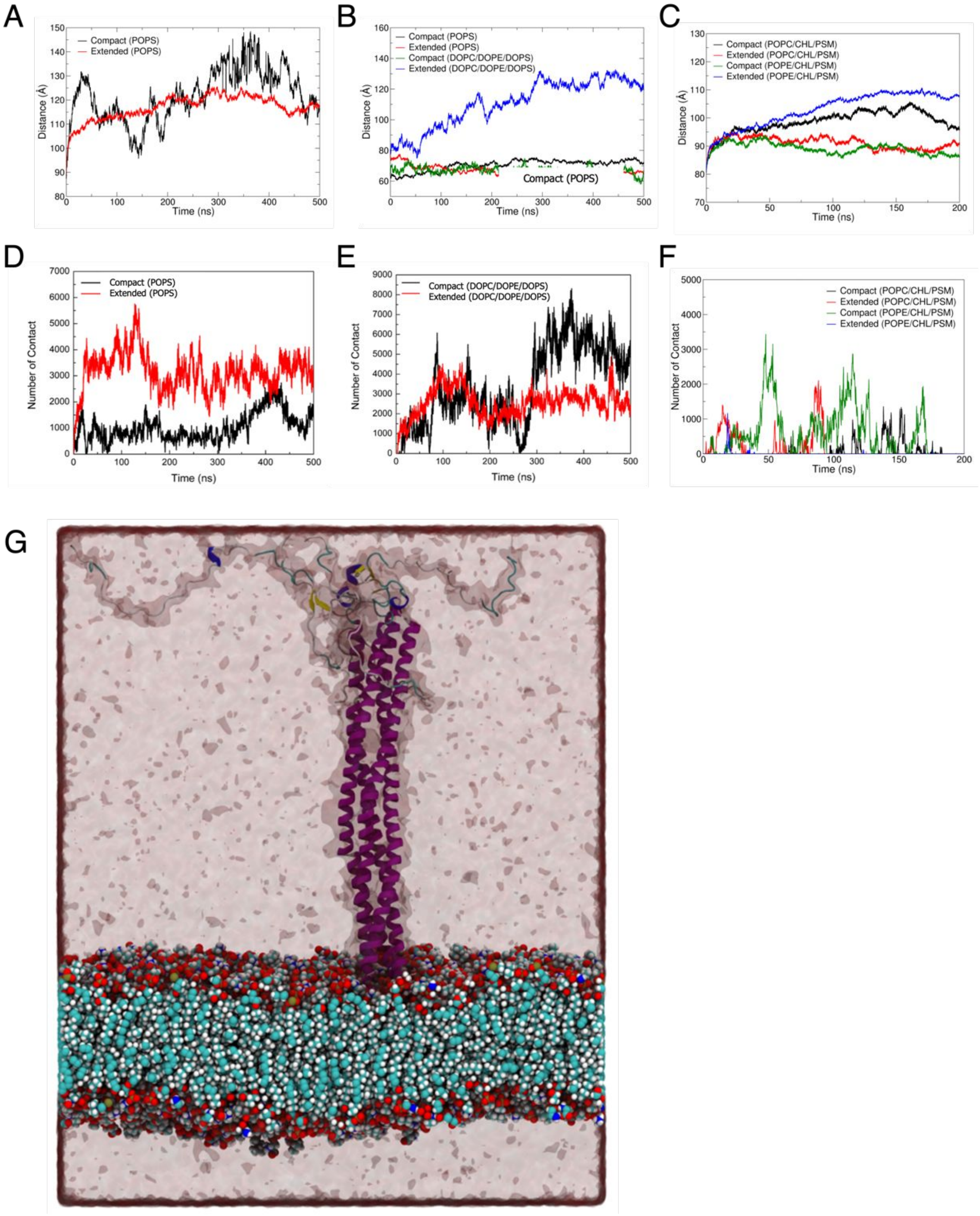

Figure S14. The change in the distance between the COM of tetramer and membrane. The initial minimum distance between tetramer and membrane is 15 Å (A) and 5 Å (B and C). The number of contacts between the tetramer and POPS membrane (D), DOPC/DOPE/DOPS membrane (E), and POPC/CHL/PSM and POPE/CHL/PSM membranes (F). The initial minimum distance between the tetramer and the membrane is 5 Å. A contact occurs if any atom of the tetramer is within 6 Å of any atom of the membrane. Note that no contact is found when the initial minimum distance between the tetramer and the membrane is set to 15 Å. (G) The simulation box of the extended αS tetramer with the DOPC/DOPE/DOPS membrane after 500 ns all-atom MD simulations. The thickness of the water layer surrounding the lipid bilayer (bottom) is about 20 Å.

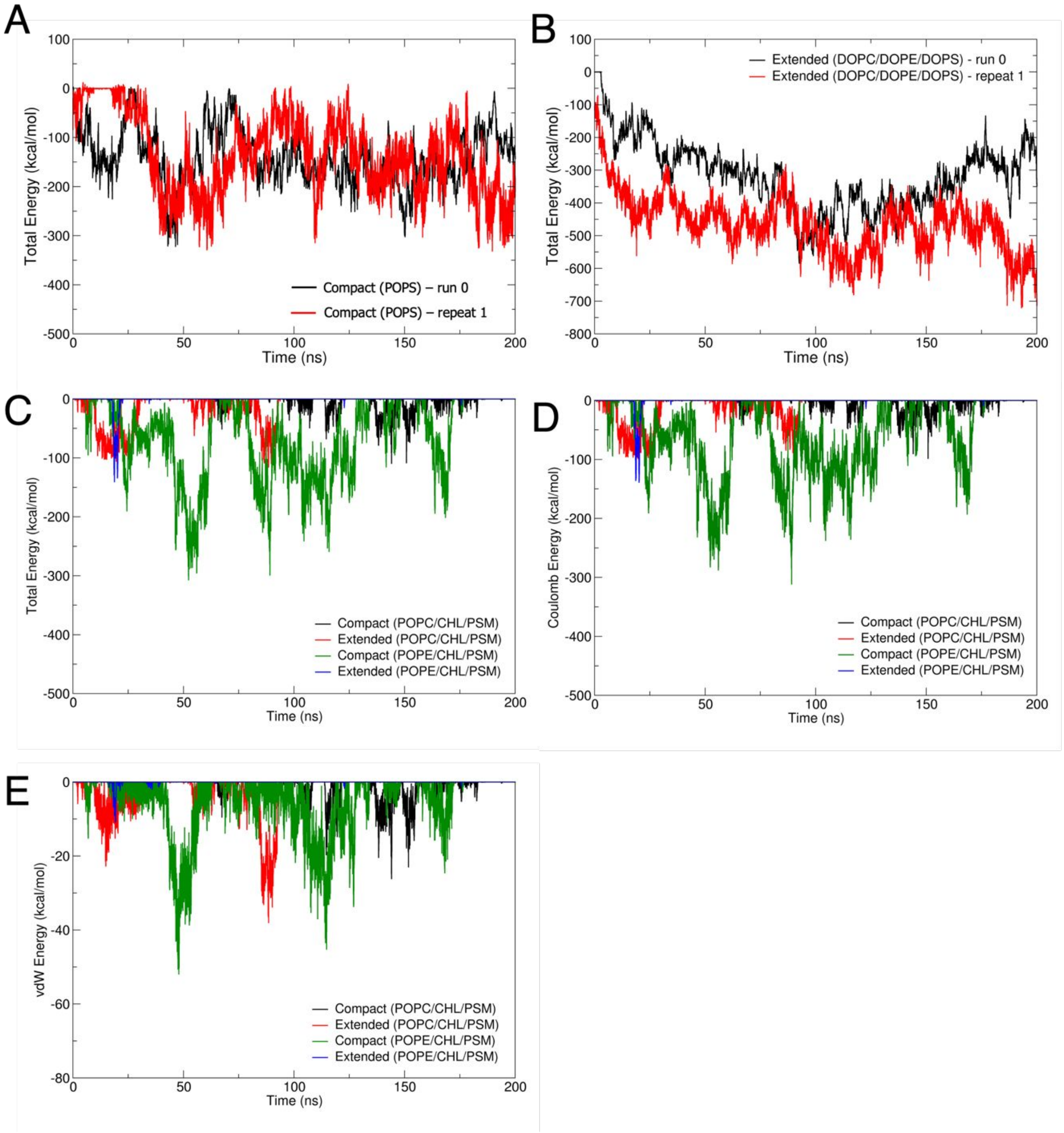

Figure S15. Comparison of the total tetramer–membrane interaction energies of initially parallel and initially tilted simulation of (A) compact helical tetramer on POPS and (B) extended helical tetramer on DOPC/DOPE/DOPS. Computed interaction energy between the tetramer and the membrane over the 200 ns MD simulations with neutral membrane types POPC/CHL/PSM and POPE/CHL/PSM (C) Total interaction energy, (D) Electrostatic interaction energy, and (E) van der Waals (vdW) interaction energy.

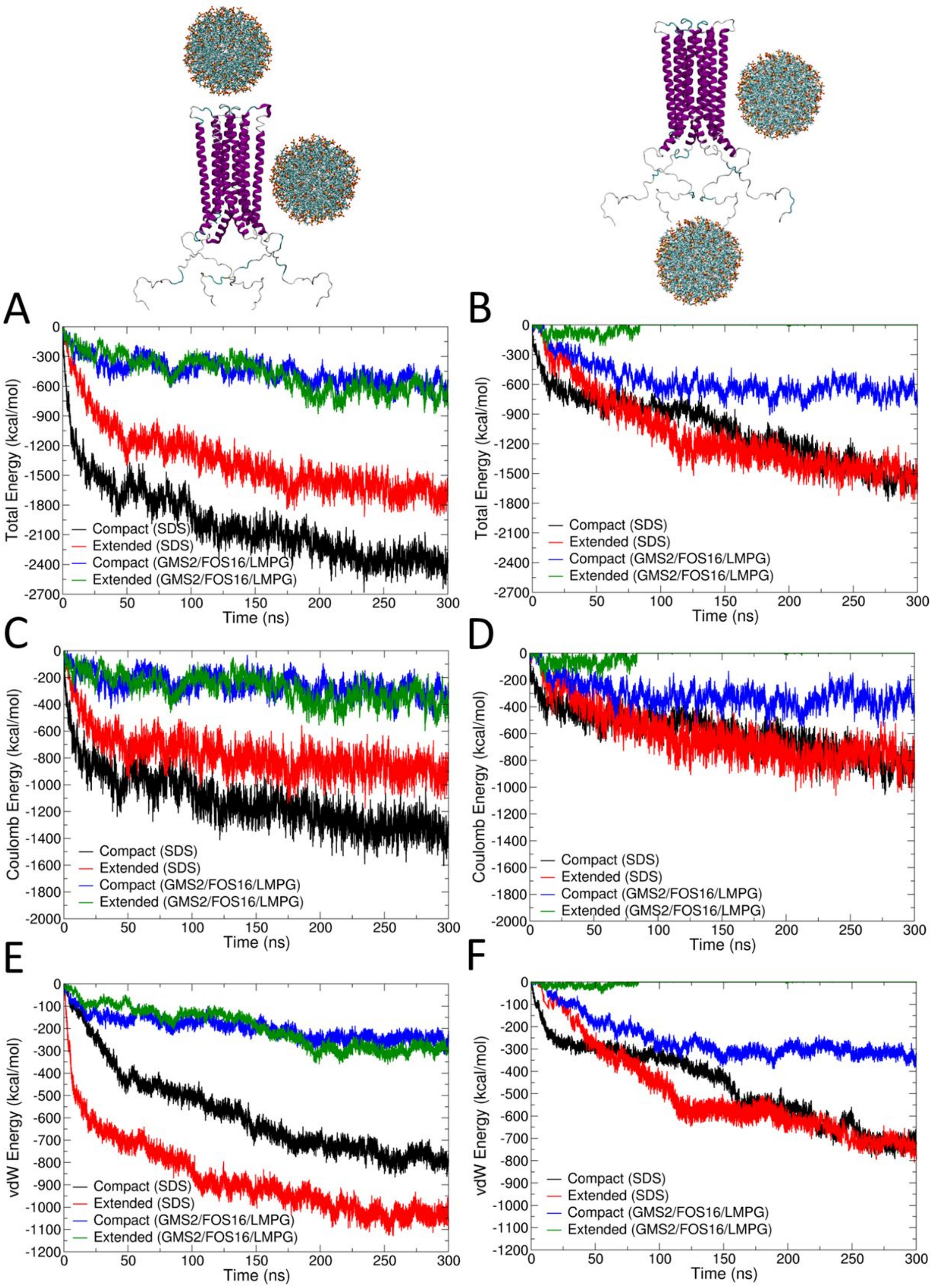

Figure S16. Computed (A, B) total, (C, D) electrostatics and (E, F) vdW interaction energies between the tetramers and the micelles with the micelles placed initially (A, C, E) at the top and the side, and (B, D, F) at the bottom and the side of the tetramer constructs.

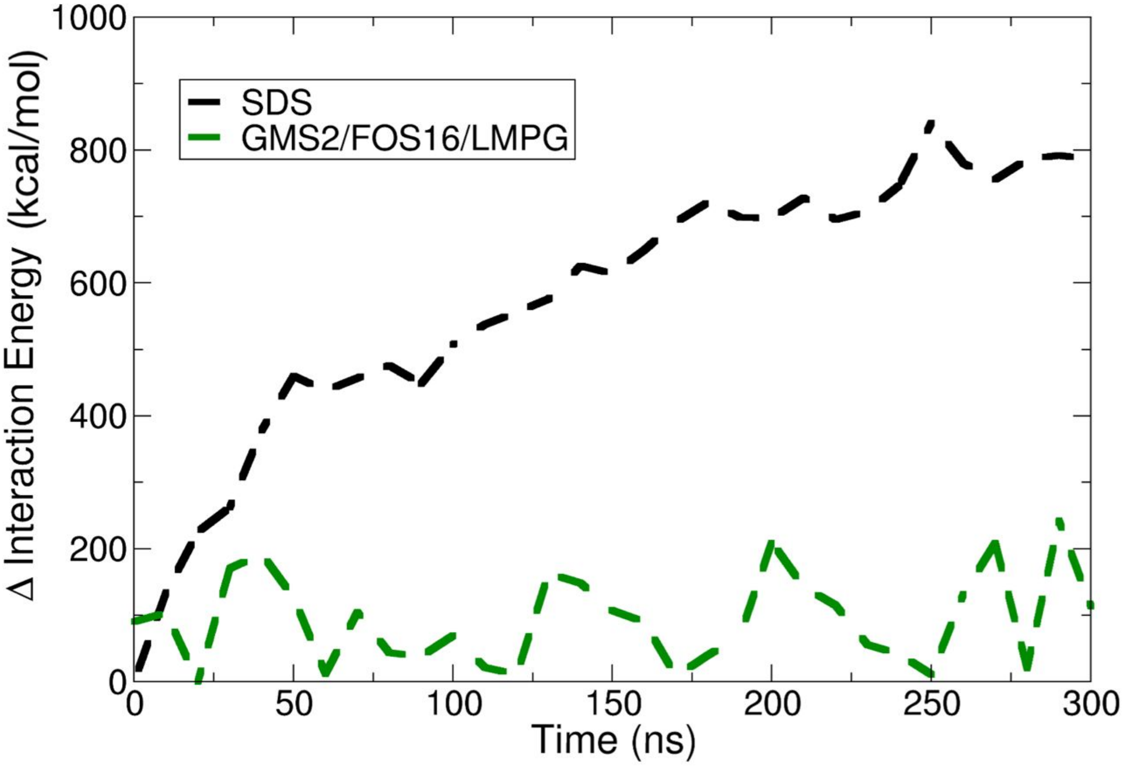

Figure S17. Plot of the difference for interaction energies between compact and extended conformations interacting with the strong negatively charged SDS micelle (black line) and with the moderately charged micelle GMS2/FOS16/LMPG (green line).

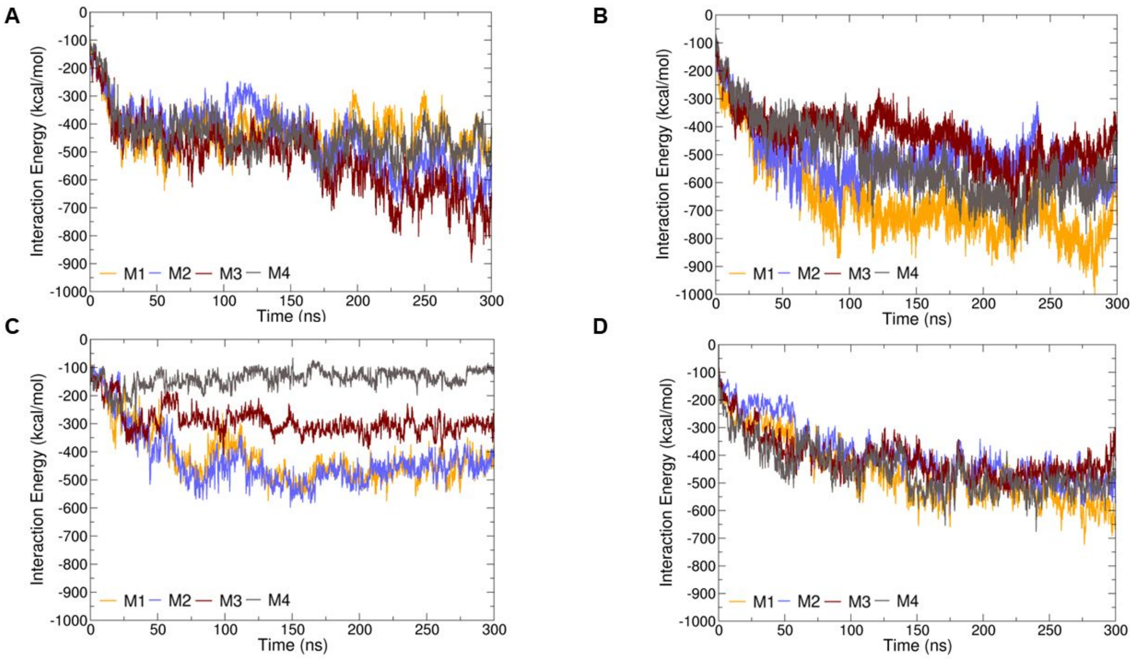

Figure S18. Total (Coulombic electrostatic + vdW energy) monomer-monomer interaction energy of the (A) compact and the (B) extended tetramer conformations in bulk water without micelles, and the (C) compact and the (D) extended tetramer conformations in presence of the SDS micelle.

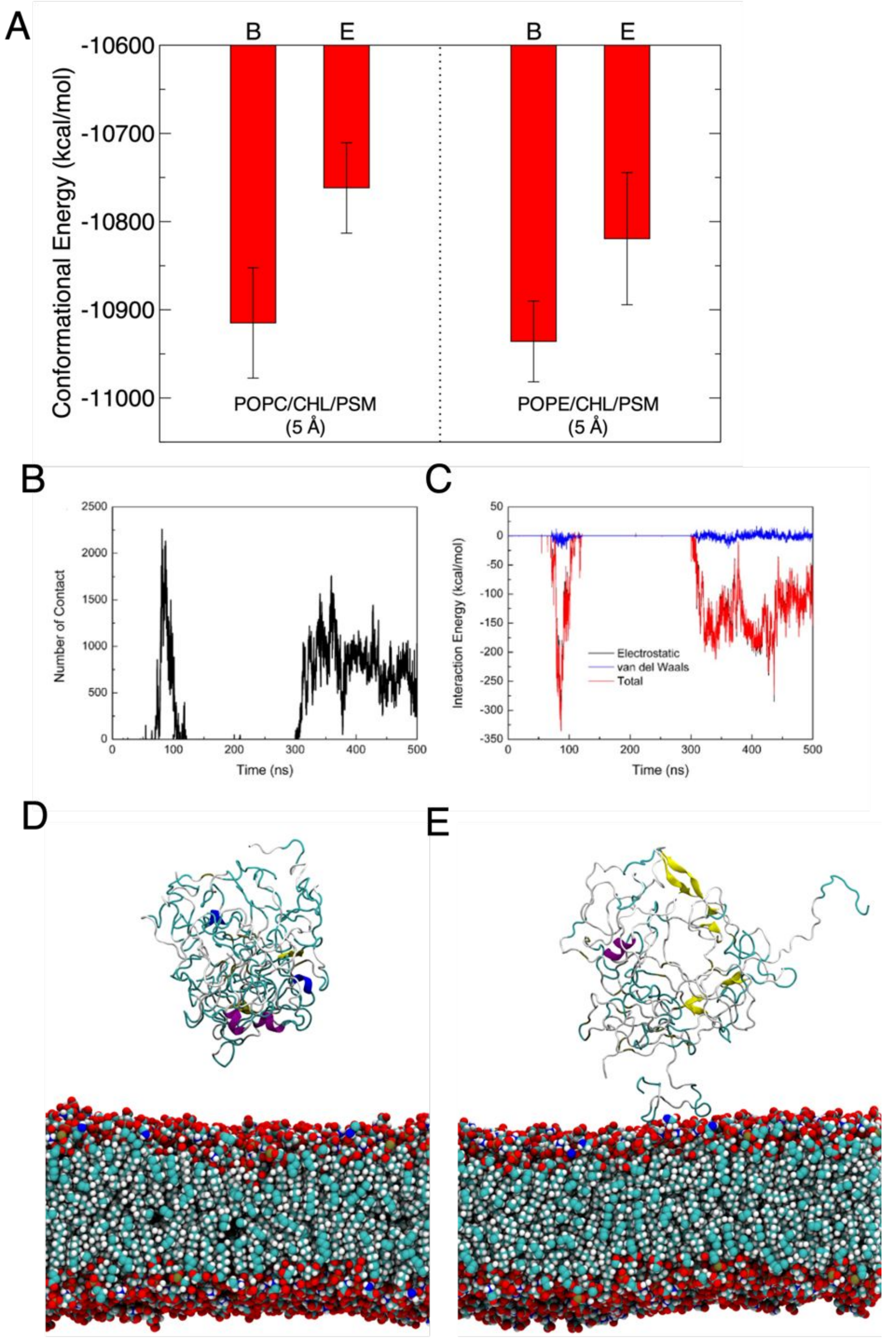

Figure S19. (A) Calculated conformation energy for the compact (denoted as B) and extended (denoted as E) helical tetramers in the presence of different neutral membranes (POPC/CHL/PSM and POPE/CHL/PSM). B-E shows results for control MD simulations of an unstructured αS tetramer on the POPS membrane. (B) The change in the number of contacts between tetramer and membrane. The initial minimum distance between the tetramer and the membrane is 15 Å, and a contact occurs if any atom of the tetramer is within 6 Å of any atom of the membrane; (C) Interaction energy between tetramer and the membrane; (D) The initial conformation with the minimum distance of 15 Å between the tetramer and the membrane surface to allow the tetramer to adopt a favourable binding orientation; and (E) the final conformation after 500 ns.

Supporting Tables

Table S1. Comparison of conformational energies of the extended helical tetramer models in water. The standard errors are shown within brackets.

Conformational energy	*Model II (kcal/mol)*	*Model III (kcal/mol)*	*Model IV (kcal/mol)*
Total	-10819 (20)	-11045 (62)	-10907 (0)
vdW	-1682 (26)	-1942 (21.8)	-1665 (22)
Electrostatic	6049 (5)	6276 (59)	3135 (72)
Solvation	-21178 (3)	-21368 (127)	-18331 (100)

Table S2. Details of the tetramer-micelle complex systems

**Tetramer conformation**	**Micelle Orientation**	**Micelle Composition**	**Short Code**
**Compact**	Bottom / Side	SDS	BSC_SDS
		GMS2/FOS16/LMPG	BSC_MIXED
	Top / Side	SDS	TSC_SDS
		GMS2/FOS16/LMPG	TSC_MIXED
**Extended**	Bottom / Side	SDS	BSE_SDS
		GMS2/FOS16/LMPG	BSE_MIXED
	Top / Side	SDS	TSE_SDS
		GMS2/FOS16/LMPG	TSE_MIXED

Table S3. Summary of conformational energies and helical percentages of two extended and one compact helical tetramer in water. The standard errors are given in brackets. ΔE_1 = energy barrier between Extended I and Compact helix models and ΔE_2 = energy barrier between Extended II and Compact helix models.

	Extended I	*Extended II*	*Compact*	*ΔE₁ (kcal/mol)*	*ΔE₂ (kcal/mol)*
Conformational energy (kcal/mol)	-11045 (62)	-10907 (0)	-11122 (3)	77	215
Helicity %	57 (0)	55 (1)	61 (1)		

Table S4. Summary of the helix percentage averaged over the last 100 ns trajectory of each system. The standard errors are shown in brackets. Note that the initial percentage of residues occupying the helical structure is about 60% and 58% in the compact and extended tetramers, respectively.

Membrane	*POPS*	
αS tetramer	Compact (initially parallel)	Extended
	59 (1)	55 (0)
	Compact (initially tilted)	
	60 (1)	
Membrane	*DOPC/DOPE/DOPS*	
αS tetramer	Compact	Extended (initially parallel)
	57 (1)	48 (1)
		Extended (initially tilted)
		56 (1)
Membrane	*POPC/CHL/PSM*	
αS tetramer	Compact	Extended
	61 (0)	55 (0)
Membrane	*POPE/CHL/PSM*	
αS tetramer	Compact	Extended
	62 (1)	59 (1)

Supporting References

1. Jao, C. C.; Hegde, B. G.; Chen, J.; Haworth, I. S.; Langen, R., *Proceedings of the National Academy of Sciences* **2008,** *105* (50), 19666-19671. DOI 10.1073/pnas.0807826105.
2. Zhang, Y.; Skolnick, J., *Nucleic Acids Res* **2005,** *33* (7), 2302-9. DOI 10.1093/nar/gki524.
3. Stetefeld, J.; Jenny, M.; Schulthess, T.; Landwehr, R.; Engel, J.; Kammerer, R. A., *Nat Struct Biol* **2000,** *7* (9), 772-6. DOI 10.1038/79006.
4. Xu, L.; Bhattacharya, S.; Thompson, D., *Chemical Communications* **2018,** *54* (58), 8080-8083. DOI 10.1039/c8cc04054k.
5. Xu, L.; Bhattacharya, S.; Thompson, D., *Physical Chemistry Chemical Physics* **2019**. DOI 10.1039/c9cp02464f.
6. Best, R. B.; Hummer, G.; Eaton, W. A., *Proceedings of the National Academy of Sciences* **2013,** *110* (44), 17874-17879. DOI 10.1073/pnas.1311599110.
